# Mountain Riparian Zones as Refugia for Rare and Endangered Plants Under Climate Change

**DOI:** 10.1002/ece3.73769

**Published:** 2026-06-01

**Authors:** Xun Lei, Mengjun Qu, Jianming Wang, Yin Wang, Wenkai Wang, Zhaoyang Wang, Baocheng Guo, Jingwen Li

**Affiliations:** ^1^ School of Ecology and Nature Conservation Beijing Forestry University Beijing China; ^2^ State Key Laboratory of Efficient Production of Forest Resources Beijing Forestry University Beijing China; ^3^ Hebei Collaborative Innovation Center for Eco‐Environment, College of Life Sciences Hebei Normal University Shijiazhuang China; ^4^ Key Laboratory of Zoological Systematics and Evolution, Institute of Zoology Chinese Academy of Sciences Beijing China

**Keywords:** biodiversity conservation, biodiversity hotspots, climate refugia, rare and endangered plants, riparian

## Abstract

Climate and land use changes pose a severe threat to plant biodiversity, particularly to rare and endangered species that are highly sensitive to environmental changes. Nevertheless, very little is understood about the spatiotemporal dynamics and current conservation status of these taxa in fragile dryland ecosystems. This study projected the potential distribution of 32 rare and endangered plant species in the Irtysh River Basin under both contemporary and projected future (2050) climate scenarios and identified biodiversity hotspots and conservation gaps based on an ensemble model that integrated Species Distribution Models (SDMs) with the InVEST habitat quality model. The results showed that water availability and topography were the primary determinants of the spatial distribution of these species, which were concentrated in riparian zones, particularly in mountainous river segments. Projections indicated that future climatic shifts would precipitate range contractions of approximately 60% of the studied species, leading to an overall decline in biodiversity across the basin. Conversely, biodiversity in mountainous areas was projected to increase, underscoring that mountainous areas acted as important climate refugia. It is worth noting that approximately 80% of the studied species were classified as gap species, highlighting severe conservation gaps in current protected area networks. These results reveal the responses of rare and endangered plants to climatic alterations and offer a sound basis for developing biodiversity conservation and management strategies in dryland ecosystems.

## Graphical abstract

This study integrates Species Distribution Models and the InVEST model to project the distribution of 32 rare and endangered plants in the Irtysh River Basin under climate change. Results predict range contractions for 60% of species by 2050 and reveal that 80% of species currently fall outside protected areas, despite the role of mountains as climate refugia. These findings offer a critical basis for optimizing conservation strategies in fragile dryland ecosystems.

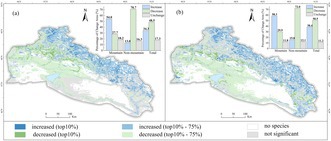

## 1 Introduction

It is widely recognized that climate and land use changes are major causes of biodiversity loss (Newbold et al. 2016; Song et al. 2021; Zhang et al. 2017). To date, persistent global warming and drastic land‐use transformations have caused extensive habitat loss and fragmentation for numerous species, especially for rare and endangered plants (Chen et al. 2011; Etard et al. 2022). These species are constrained by diminutive population sizes, impoverished genetic diversity, and narrow ecological niches, having a severely limited capacity to adapt to or buffer against environmental perturbations (Dee et al. 2019; Vincent et al. 2020). Worse still, the risks of habitat degradation and extinction for endangered plants have been further exacerbated by the simultaneous progress of global warming and human activity intensification in recent years (Schwartz et al. 2006). Therefore, conserving rare and endangered plants is not only essential for safeguarding biodiversity but also for maintaining the structural and functional integrity of ecosystems, and assessing the impacts of climate change on these species is of non‐negligible importance.

Mountains play a critical role in mediating plant responses to climate change (Elsen and Tingley 2015; Pinkert et al. 2025; Zhou et al. 2023). Mountainous areas, which are characterized by pronounced topographic heterogeneity and intricate microenvironments, serve simultaneously as biodiversity hotspots and critical refugia for species (Brighenti et al. 2021; Ding et al. 2020; Keppel et al. 2012). The sharp environmental gradients inherent to mountainous areas enable flora to alleviate the impact of climate warming and track suitable climatic niches through relatively short‐distance upslope migrations, thereby effectively reducing extinction risks (Chan et al. 2024; Mahmoodi et al. 2023). Conversely, species in non‐mountainous areas are frequently compelled to traverse extensive geographic distances to adapt themselves to shifting climatic regimes (Ackerly et al. 2010). Consequently, a comprehensive understanding of the differential responses of rare and endangered plants in mountainous versus non‐mountainous areas is of significant importance for identifying priority conservation areas.

Species Distribution Models (SDMs) are extensively used to project potential species distributions and assess range‐wide responses to climatic variations (Elith and Leathwick 2009; Pillet et al. 2022). These models are commonly employed to evaluate extinction risks for rare and endemic taxa and to delineate biodiversity hotspots and priority conservation areas (Gallardo et al. 2017; Huang et al. 2020; Li et al. 2021; Ye et al. 2021). However, conventional SDMs predominantly emphasize climatic suitability and frequently neglect the constraints imposed by actual land use and land cover (LULC) conditions on species survival (Wang et al. 2024). To mitigate this limitation, the integration of SDM projections with the Integrated Valuation of Ecosystem Services and Tradeoffs (InVEST) model emerges as a critical pathway for comprehensive assessment (Men and Pan 2025; Yang et al. 2025). Specifically, when coupled with SDMs projections, evaluating habitat quality via the InVEST model based on LULC data facilitates the precise identification of biodiversity hotspots and conservation gaps (Huang et al. 2020).

The Irtysh River Basin is not only a water conservation area and an important ecological barrier in Central Asia but also a key biodiversity hotspot in arid and semi‐arid regions (Liu et al. 2021; Xu et al. 2024). Despite its significant ecological strategic importance, the risk of habitat fragmentation for rare and endangered plants in this region has not been sufficiently quantified, and there remain uncertainties regarding whether current conservation networks can adequately address future climate uncertainties. To fill the knowledge gap, this study used 32 typical rare and endangered plant species in the basin as research subjects and predicted their responses to climate change based on an ensemble model that integrated Species Distribution Models (SDMs) with the InVEST habitat quality model. The specific objectives of this study included: (1) predicting the potential distribution range of rare and endangered plants under current and future climate scenarios; (2) identifying biodiversity hotspots for these species; (3) identifying conservation gaps and priority protection areas for biodiversity conservation and management.

## 2 Materials and Methods

### 2.1 Study Area

**FIGURE 1:**
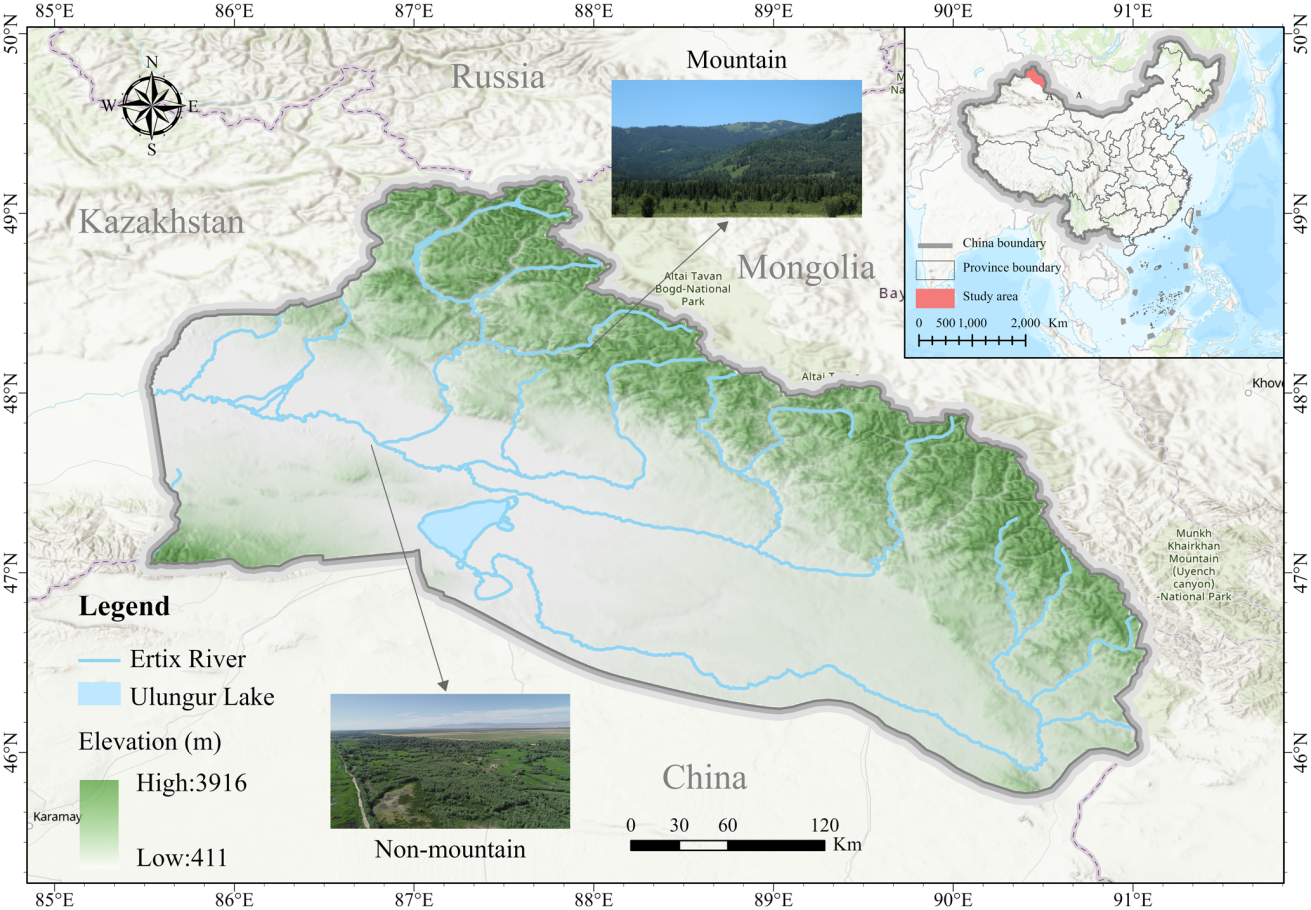
Location and topography of the Irtysh River Basin.

The Irtysh River Basin is located in the Altay Prefecture of northern Xinjiang, China (85°31′–91°04′ E, 45°00′–45°10′ N), which lies in the heart of the Eurasian continent and is characterized by a temperate continental climate (Figure 1). The Irtysh River originates from the Altai Mountains, with a length of approximately 633 km and a drainage basin area of 45,300 km^2^ (Liu et al. 2022). Precipitation in the basin is highly unevenly distributed, with annual averages reaching up to 500 mm in the mountainous areas and 150 mm in the plains. The geomorphological units are clearly delineated, including the northern mountainous region, the central hills and fluvial plains, and the southern deserts and Gobi, accounting for 32%, 22%, and 46% of the basin, respectively (Liu et al. 2022; Wang et al. 2016). The combination of abundant water resources and distinctive topographic and geomorphological features makes the basin one of the most famous biodiversity hotspots, harboring numerous endangered and rare plant species, such as *Prunus tenella* Batsch and *Cistanche deserticola* Ma.

However, climate and land use changes have collectively led to vegetation degradation in the Irtysh River Basin, severely threatening the survival of rare and endangered plant species (Luo et al. 2022). Therefore, the influence of climate and land use changes should be taken into account in the prediction of current and future biodiversity hotspots.

### 2.2 Data

#### 2.2.1 Species Occurrence Data

To identify the rare and endangered species to be included in the analysis, we consulted the List of National Key Protected Wild Plants of China (https://www.gov.cn/zhengce/zhengceku/2021‐09/09/content_5636409.htm), the List of Key Protected Wild Plants of the Xinjiang Uygur Autonomous Region (https://lcj.xinjiang.gov.cn/lcj/zcwj/202401/a2a9c2b4dcaf4174b4625e4f5331cd8e.shtml), and the IUCN Red List of Threatened Species (https://www.iucnredlist.org).

Distribution records of endangered plant species were obtained during three consecutive years of field investigations (2022–2024). To ensure the authenticity and comprehensiveness of the data, the design of the survey routes adhered to the following principles: (1) encompassing potential distribution sites documented in existing literature and online data‐sharing platforms (e.g., GBIF, CVH); and (2) covering all representative ecosystem types in the basin. Ultimately, a total of 1068 valid occurrence records, representing 66 endangered plant species, were collected (Appendix S1: Table S1).

To mitigate overfitting in the ecological niche models, data preprocessing was conducted using the R‐4.3.3 “raster” package (Hijmans 2025a). Specifically, only a single occurrence record was retained within each 0.1° × 0.1° grid cell, and species with fewer than five records were excluded. Following this procedure, a final dataset comprising 32 species and 620 occurrence records was obtained for simulating the spatial distribution of rare and endangered wild plants under future climate scenarios.

#### 2.2.2 Environmental Variables

A total of 24 environmental variables were involved in this study, including 19 bioclimatic factors, three topographic factors, and two distance factors, which may potentially influence the distribution of rare and endangered plant species. Due to differences in spatial resolution among the original environmental variable layers, all environmental variables were uniformly resampled to a 30‐arc‐s (~1 km) spatial resolution to ensure consistency in spatial analyses. Details regarding data sources and processing are provided in Table 1.

**TABLE 1:** Environmental variables.

Variables	Indicators	Data sources	Data processing
Bioclimatic variables	Bio1‐Bio19	WorldClim (https://www.worldclim.org/)	Current (the average for the years 1970–2000) and future (2050) bioclimatic variables for the SSP245 climate change scenario were selected from the BCC‐CSM2‐MR GCM
Topographic dataset	DEM, Slope, Aspect	Geospatial Data Cloud Platform (https://www.gscloud.cn)	Slope, aspect, and elevation data were extracted from the ASTER GDEM V2 digital elevation model (DEM) data through the ArcGIS Pro
Distance factors	Disriver, Dislake	Resource and Environmental Science Data Platform (https://www.resdc.cn/)	The distance to rivers and lakes were calculated using Euclidean distance by ArcGIS Pro
Land use	Current and future land use types	Resources and Environmental Science Data Center (http://www.resdc.cn/)	Based on three‐period land use data from 2000 to 2020 at a 1 km resolution, the PLUS model was employed to forecast future land use types (for the year 2050). The detailed methodology is provided in the Supporting Information

To ensure a high prediction accuracy of the model, all environmental variables were subjected to Variance Inflation Factor (VIF) and Pearson correlation analyses using the “usdm” package in R software (Pearson et al. 2007; Salmerón Gómez et al. 2016). Environmental variables with Pearson correlation coefficients below 0.8 and VIF values < 10 were selected for the subsequent construction of species distribution models.

### 2.3 Coupled Modeling Framework

We developed a coupled modeling framework that integrated SDMs with the InVEST model. In this framework, SDMs were first used to project the potential distribution of rare and endangered plant species under current and future scenarios, and the InVEST model was then used to evaluate habitat quality (Natural Capital Project 2022). The integration of these two components enabled the identification of biodiversity hotspots and conservation gaps by jointly considering species suitability and habitat condition.

#### 2.3.1 Species Distributions Model

We predicted the potential distribution range of rare and endangered plants under current and future climate scenarios using the following eight algorithms within the “biomod2” R package (Thuiller et al. 2009): generalized additive models (GAM), flexible discriminant analysis (FDA), classification tree analysis (CTA), extreme gradient boosting (XGBoost), surface range envelope (SRE), random forests (RF), multivariate adaptive regression splines (MARS), and maximum entropy (MaxEnt).

To calibrate and evaluate the models, 1000 pseudo‐absence points were generated for each species using a random sampling approach. The dataset was then partitioned via cross‐validation, with 75% used for model training and 25% reserved for validation. This procedure was repeated ten times to enhance model robustness. Model accuracy was assessed using the area under the receiver operating characteristic curve (AUC) and the true skill statistic (TSS). AUC values range from 0 to 1, with values closer to 1 indicating superior model fit and performance (Peterson et al. 2008), while TSS values range from −1 to 1, with values approaching 1 reflecting higher predictive accuracy (Allouche et al. 2006).

Following the evaluation of individual models, ensemble models were constructed using a weighted‐mean approach. Specifically, only individual models with AUC values exceeding 0.9 and TSS values > 0.8 were incorporated into the ensemble, thereby reducing the influence of poorly performing models, limiting dependence on any single algorithm, and minimizing overall model uncertainty (Araújo and New 2007; Breiner et al. 2015; Marmion et al. 2009; Thuiller et al. 2009). Finally, species suitability was converted into binary presence–absence data (0 = absence, 1 = presence) based on the maximum TSS threshold for subsequent analyses (Barbet‐Massin et al. 2012).

#### 2.3.2 InVEST Prediction

The InVEST Habitat Quality model was used to assess habitat quality across the study area based on LULC data, threat‐factor parameters, and the sensitivity of different LULC types to biodiversity threats. Based on previous studies (Lu et al. 2024; Wang and Cheng 2022), the threat factors (Table S2) and sensitivity parameters (Table S3) used in the model were defined. The projected LULC map for 2050 was derived from the PLUS model. Detailed procedures for the PLUS simulation are provided in the Appendix S2. Habitat quality was calculated for each raster cell as follows:

(1) $$D_{\mathrm{xj}}=\sum_{r=1}^{R}\sum_{y=1}^{Y_{r}}\left(\frac{w_{r}}{\sum_{r=1}^{R}w_{r}}\right)r_{y}i_{\mathrm{rxy}}\beta_{x}S_{\mathrm{jr}}$$

(2) $$\begin{array}{c} Q_{\mathrm{xj}}=H_{j}\times \left[1-\left(\frac{D_{\mathrm{xj}}^{z}}{D_{\mathrm{xj}}^{z}+k^{z}}\right)\right] \end{array}$$

(3) $$\begin{array}{c} i_{\mathrm{rxy}}=1-\left(\frac{d_{\mathrm{xy}}}{d_{\text{rmax}}}\right) \end{array}$$

(4) $$\begin{array}{c} i_{\mathrm{rxy}}=\exp\left(-\frac{2.99d_{\mathrm{xy}}}{d_{\text{rmax}}}\right) \end{array}$$

where $D_{\mathrm{xj}}$ is the habitat degradation score of grid cell a with LULC type *j*; $Y_{r}$ is the set of grid cells on the raster of threat *r*; $r_{y}$ indicates the intensity of threat *r* in grid cell *y*;$W_{r}$ is the weight of threat *r*; $\beta_{x}$ is the accessibility of grid cell *x*; $S_{\mathrm{jr}}$ is the sensitivity of LULC type *j* to threat *r*; and $i_{\mathrm{rxy}}$ represents the impact of threat *r* from grid cell *y* on grid cell a as a function of distance. $Q_{\mathrm{xj}}$ is the habitat quality score of grid cell a with LULC type *j*, ranging from 0 to 1; $H_{j}$ is the habitat suitability of LULC type *j*; and *k* is the half‐saturation constant. In addition, $d_{\mathrm{xy}}$ is the linear distance between grid cells and *y*, and $d_{\text{rmax}}$ is the maximum effective distance over which threat *r* exerts an influence.

### 2.4 Data Analysis

#### 2.4.1 Quantifying Species Responses

To evaluate how future climatic conditions may affect the geographic ranges of 32 rare and endangered plant species, we calculated each species' potential distributional area under both current and projected climate scenarios using the “terra” package in R (Hijmans 2025b). We used the percentage change in climatically suitable habitat (CSH) as the key metric for quantifying species' distributional responses, computed as follows:

(5) $$\begin{array}{c} \mathrm{CSH}=\frac{{\text{Area}}_{\text{Future}}-{\text{Area}}_{\text{Current}}}{{\text{Area}}_{\text{Current}}}\times 100 \end{array}$$

where Area_Current_ and Area_Future_ are the areas of current and future habitats. This formula estimates the extent of range expansion or contraction, with positive values indicating increased suitable habitat area and negative values indicating a decrease.

#### 2.4.2 Biodiversity Analyses

To evaluate the biodiversity of rare and endangered plants, this study selected two indicators: species richness (SR) and phylogenetic diversity (PD). Species richness (SR) is a fundamental indicator for measuring biodiversity, calculated as the total number of species within a given spatial unit (e.g., grid cell) (Gotelli and Colwell 2001; Whittaker 1972), and we computed and mapped SR by integrating binary distribution maps of each species.

Phylogenetic diversity (PD) refers to the total evolutionary history represented by a given taxonomic group (Faith 1992; Tucker et al. 2017), reflecting the evolutionary differences and uniqueness among species (Faith 2013; Winter et al. 2013). Evaluating phylogenetic diversity plays an irreplaceable role in understanding functional diversity (Owen et al. 2019) and developing biodiversity conservation strategies (Faith 2021). We constructed a phylogenetic tree for 32 rare and endangered plants based on plant taxonomic information using the “V.PhyloMaker2” package (version 0.1.0) in R (Jin and Qian 2019), and quantified phylogenetic diversity by calculating the cumulative sum of phylogenetic branch lengths for all species within grid cells using the “picante” package (version 1.8.2) in R (Faith 1992; Kembel et al. 2010).

Biodiversity hotspot identification is one of the key approaches for biodiversity conservation and typically uses the top 2.5%, 5%, or 10% of high‐value areas within a study region as the hotspot threshold (Huang et al. 2016; Orme et al. 2005; Zhu et al. 2023). Numerous studies have demonstrated that adopting the top 5% threshold provides the optimal approach for identifying biodiversity hotspots (Huang et al. 2016; Pinkert et al. 2025). Accordingly, this study defined biodiversity hotspot areas as those falling within the top 5% in species richness (SR), phylogenetic diversity (PD), and habitat quality (HQ). This integrated definition not only captures both intra‐ and interspecific diversity patterns of rare and endangered plant species with greater precision (Voskamp et al. 2023) but also effectively avoids the overestimation of results that may arise from neglecting land‐use conditions (Wang et al. 2024).

#### 2.4.3 Centroid Shift Analysis

To quantify the migratory dynamics of rare and endangered plant species under climate change, this study performed centroid analysis using the “geosphere” package in R (Hijmans 2024). By comparing centroid positions, we determined the magnitude and direction of range shifts. In addition, the “sf” package was used to generate standard ellipses, thereby further confirming the direction of range shifts.

#### 2.4.4 Identifying Priority Conservation Areas

To identify priority conservation areas in the Irtysh River Basin, a comprehensive prioritization framework was developed. Specifically, we identified several hotspot areas (top 10% for SR, PD, and HQ) and high‐risk areas (top 10% of projected species loss) across the basin. Using spatial overlay analysis, we designated the intersections of layers as priority areas for conservation.

We overlaid the priority conservation areas of rare and endangered plant species with the map of China's nature reserve (PA) and identified the portions lying outside the PA boundaries as conservation gaps. Data on China's nature reserves were obtained from the National Specimen Information Infrastructure (https://www.nsii.org.cn/2017/home.php).

## 3 Results

### 3.1 Model Accuracy Evaluation

The TSS values of the ensemble model (EM) ranged up to 1, with most exceeding 0.9 (Figure S1), indicating that the model had excellent performance in projecting the spatial distribution patterns of rare and endangered plant species in the Irtysh River Basin.

### 3.2 Changes in Rare and Endangered Plants Under Climate Scenarios

**FIGURE 2:**
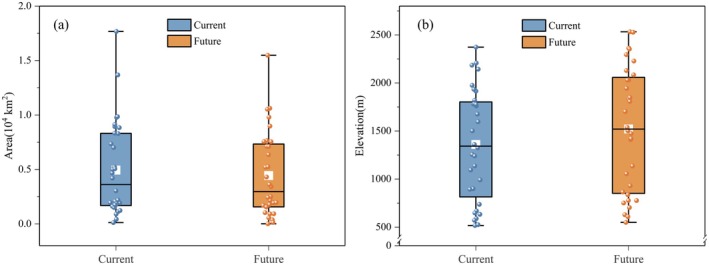
Projected changes in suitable habitat area (a) and elevation (b) for 32 rare and endangered plant species under future climate change by 2050.

The potentially suitable habitat for the 32 rare and endangered plant species had an average area of 0.49 × 10^4^ km^2^ and an average elevation of 1359.65 m under current climate scenarios. However, projections showed that as a result of future climate change, there would be an average 10.20% reduction in suitable habitat area, accompanied by an 11.86% upward shift in mean elevation (Figure 2).

**FIGURE 3:**
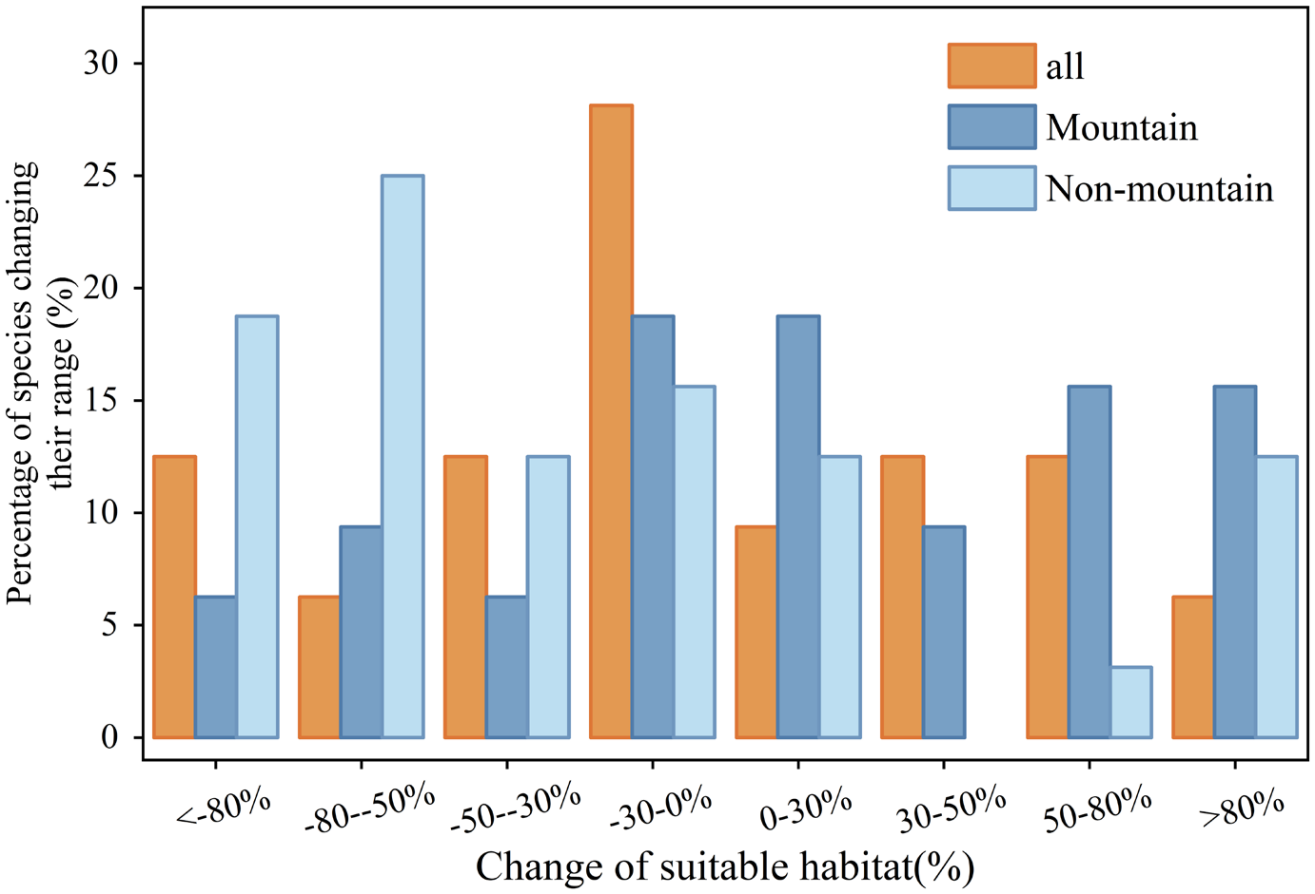
The percentage of rare and endangered plant species changing habitat suitability (CSH) under future climate change.

In addition, projections indicated that 40.62% of the studied species (13 species) would experience range expansions (CSH > 0), while the others (19 species) would experience range contractions (CSH < 0) (Figure 3). Specifically, non‐mountainous species were expected to predominantly contract their suitable range, with a disproportionately high number of them suffering a drastic range shrinkage (CSH ≤ −50%) (Figure 3). In contrast, mountainous species were more likely to expand their suitable range, with a large proportion of them showing a slight range expansion (CSH = 0%–30%) (Figure 3 and Figure S2).

**FIGURE 4:**
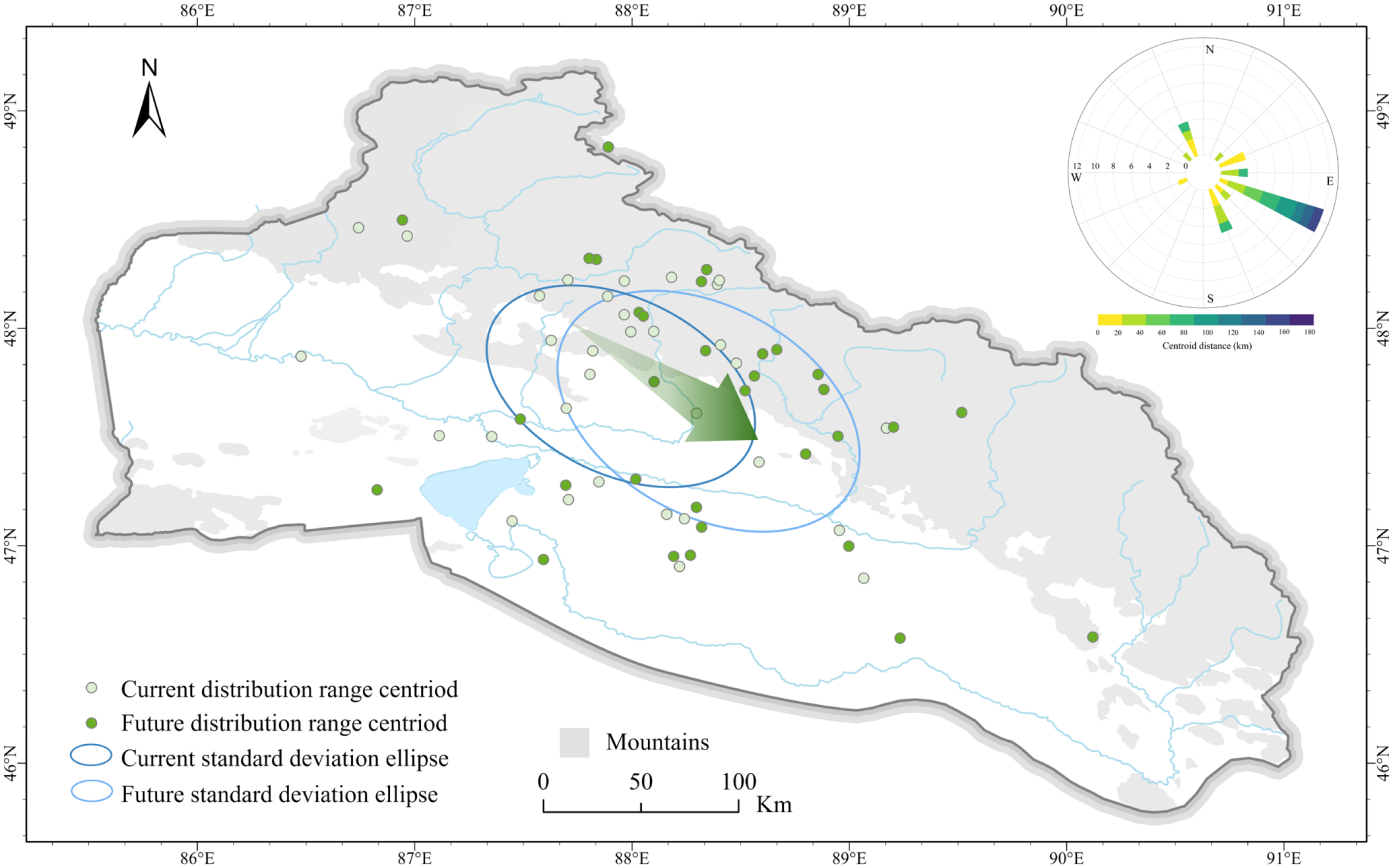
Geographic centroid shift of rare and endangered species under future climate scenarios. The inset polar coordinate diagram illustrates migration distance and direction for species. Colors indicate migration distance; numbers denote the number of species.

Moreover, projections showed that among the studied species, those migrating southeastward were expected to constitute the majority and accounted for the greatest mean migration distance. In contrast, only a few species would migrate northwestward, southwestward, and northeastward (Figure 4). Overall, future climate change was projected to drive a systematic displacement of species' geographic centroids from western mountainous regions toward eastern mountain ranges, and from higher latitudinal regions toward lower latitudinal regions. In comparison, the centroids of the major climatic variables were projected to shift northwestward across the basin under future scenarios (Figure S3). This contrast suggests that the projected species redistribution cannot be explained simply as spatial tracking of basin‐scale macroclimatic shifts.

### 3.3 Spatial Patterns of Change in Diversity

**FIGURE 5:**
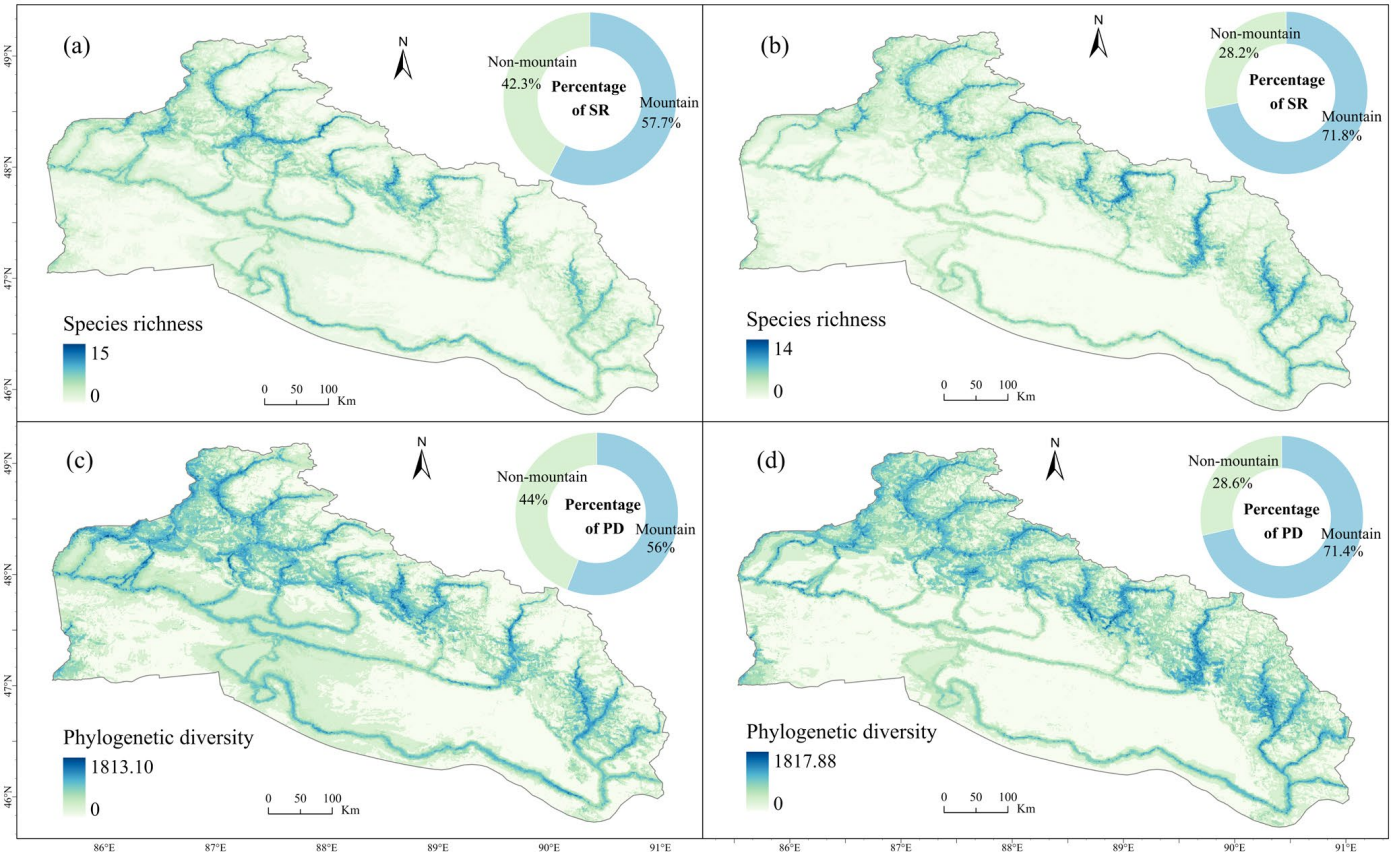
Spatial distribution of rare and endangered plant diversity under current and future climate scenarios. The left panels (a, c) illustrate the current climate scenario, while the right panels (b, d) depict the future climate scenario. (a, b) Species richness; (c, d) Phylogenetic diversity.

Riparian zones along both sides of the major rivers consistently represented the core areas of SR and PD (Figure 5), harboring the majority of biodiversity in the basin under both current and future climate scenarios, and the upper reaches of the Burqin River had the highest SR and PD under current climatic conditions (Figure 5a,c). Although overall diversity was projected to decline in the future, the relative contribution of mountainous species to both SR and PD would increase substantially (Figure 5b,d).

**FIGURE 6:**
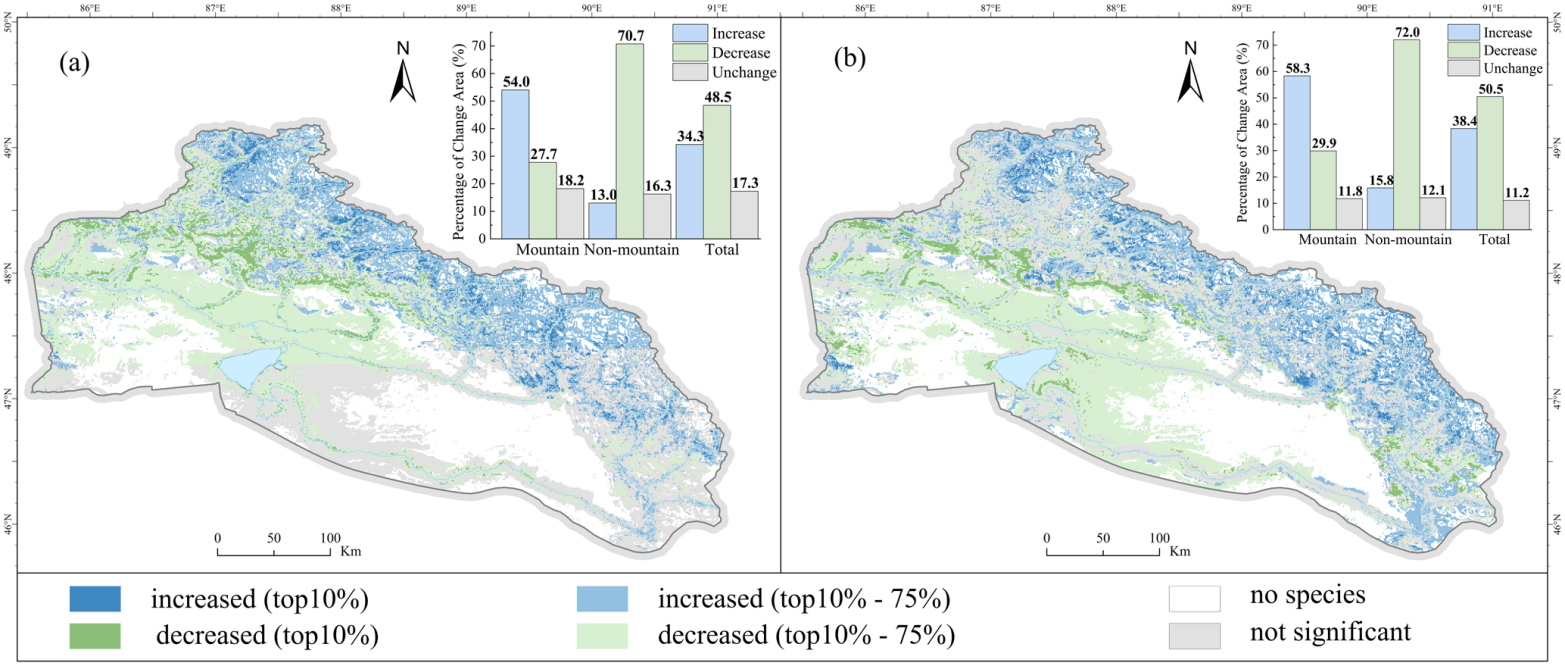
Projected spatial changes in rare and endangered species diversity under future climate change. (a) Species richness; (b) Phylogenetic diversity. The bar chart displays the percentage of grid cells where SR and PD show increasing, decreasing, or unchanged trends in mountainous, non‐mountainous, and overall regions.

Projections showed that the distribution patterns of SR and PD would exhibit similar trends in response to future climate change. Specifically, approximately 70.74% and 72.03% of the downstream non‐mountainous areas were projected to exhibit a decrease in SR and PD, respectively, with the most pronounced decreases occurring in the lower riparian areas of the Burqin River. In contrast, upstream mountainous areas were projected to show an increase in SR and PD, with the most significant growth occurring around Kanas Lake and the Qinghe region (Figure 6).

### 3.4 Predicting Hotspots of Rare and Endangered Species

Habitat quality showed clear spatial heterogeneity across the Irtysh River Basin (Figure S4). Areas with relatively high HQ were mainly distributed in remote mountainous regions, whereas lower HQ values were concentrated in non‐mountainous areas that were more heavily affected by human activities.

**FIGURE 7:**
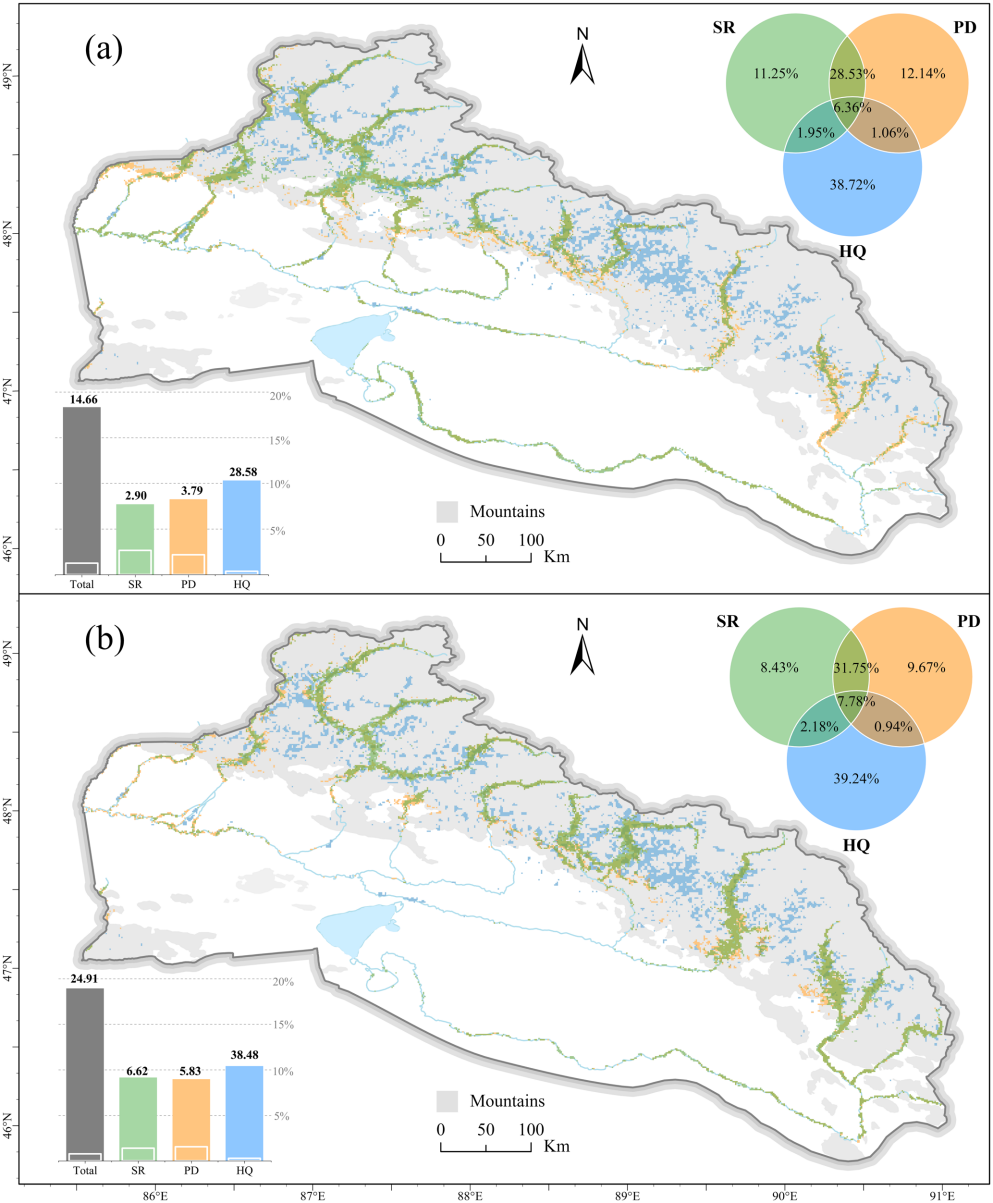
Hotspots for rare and endangered species under current (a) and future (b) conditions. The inset Venn diagram illustrates the percentage overlap among the three types of hotspots—SR (green), PD (orange), and HQ (blue). The inserted bar chart displays the probability of hotspots occurring in mountainous areas (solid bars) versus non‐mountainous areas (white outlines), integrating all aspects—Total (gray), SR (green), PD (yellow), and HQ (blue). The values above the bars indicate the ratio of these two probabilities.

By integrating SR, PD, and HQ, we identified biodiversity hotspots for rare and endangered plant species across the basin. The total area of these hotspots was 0.44 km^2^, accounting for 5.36% of the entire study area. These hotspots were concentrated along the banks of main and tributary rivers across the basin, forming core clusters in mountainous river sections (Figure 7). Among the three types of hotspots, SR and PD hotspots shared the most pronounced overlap. Moreover, a comparison between current and future scenarios indicated a notable spatial shift in hotspot distribution. Under current climatic conditions, hotspots were predominantly situated in the western portion of the study area, particularly in the upper reaches of the Burqin River. However, future climate change would cause an eastward shift in hotspot distributions, specifically to the upper reaches of the Kara Irtysh River and the Irtysh River.

### 3.5 Priority Conservation Areas

There were seven protected areas in our study area, including three national‐level and four provincial‐level nature reserves (Table S4). These reserves covered a total area of 10,481.4 km^2^, accounting for 12.87% of the study area. However, the existing protected area network only encompassed a small fraction of ecologically important zones. Gap species (with conservation coverage of 0%–20%) constituted 81.25% (26/32) of the studied species. Among them, five species—including *Cynomorium songaricum*, *Ephedra intermedia*, and *Tulipa heteropetala*—were particularly underprotected, each with conservation coverage below 5% (Table S5). Notably, the distribution range of *Tulipa biflora* lay entirely outside the existing protected areas.

**FIGURE 8:**
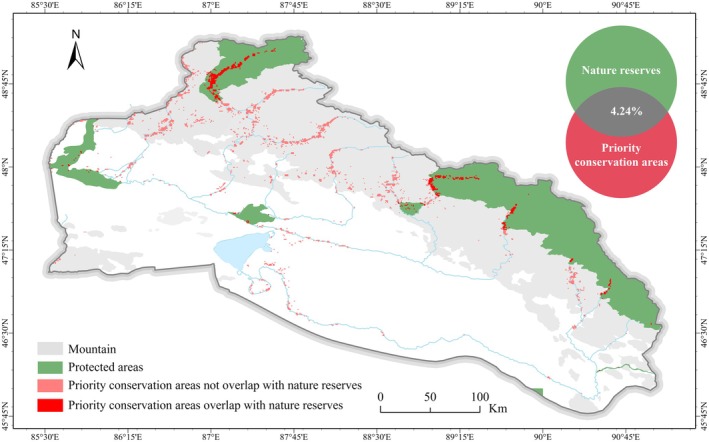
Priority conservation areas for rare and endangered plants. Gray represents mountains, green represents protected area.

Under current climatic and land‐cover conditions, the identified priority conservation areas only shared a 4.24% overlap with the existing nature reserves. Priority conservation areas were concentrated in the upper reaches of the mountainous tributaries, such as the Burqin River, Haba River, and Kran River. In contrast, only a few priority conservation areas were distributed along the main rivers, where intensified human disturbance posed a severe threat to biodiversity, necessitating enhanced protection measures (Figure 8).

## 4 Discussion

### 4.1 Changes in Future Rare and Endangered Plants

Numerous studies have demonstrated that climate and land use changes exert a significant influence on species richness and phylogenetic diversity (Hu et al. 2024; Pinkert et al. 2025; Wu et al. 2025). In particular, rare and endangered plants in arid regions are found to exhibit heightened sensitivity to climate change (Elsen and Tingley 2015). Our results also predicted that approximately 60% of rare and endangered plant species would experience a range contraction in the future, leading to decreased biodiversity (SR and PD) across the Irtysh River Basin. These findings align with the predictions of species distribution models that because of climate change there will be a decrease in the diversity of rare and endangered plants in arid regions (Dong et al. 2024). As a result, future climate warming is widely believed to be the primary cause for the range contractions of rare and endangered plants, considering their narrow geographic distribution and exceptional sensitivity to climate change (Dolezal et al. 2021).

However, not all species are projected to suffer adverse impacts from future climate change. In this study, 13 plant species were anticipated to undergo range expansions, which may be attributed to a difference in niche breadth. In arid regions, increased precipitation can alleviate water scarcity and enhance habitat suitability for plants, thereby facilitating their range expansions (Lian et al. 2021; Wang et al. 2018). Additionally, climate change may enhance the survival and reproductive capacities of certain species, such as *Xanthoceras sorbifolium* Bunge (Guo et al. 2023).

Furthermore, we observed that rare and endangered plants in the Irtysh River Basin would shift toward higher elevations, consistent with the global migration patterns of mountainous plants (Chen et al. 2025). The rising temperatures render temperature‐sensitive species more inclined to migrate to higher elevations (Figure S5) (Chen et al. 2011). Simultaneously, human activity is more intense in lowland river valleys, and land use changes significantly impact plant distributions.

This study found that rare and endangered plant species would predominantly migrate southeastward in response to climate change, contradicting a previous study that reported a northwestward migration (Sun et al. 2025) and diverging from the northwestward shift of the basin's macroclimatic centroids (Figure S3). We hypothesize that this discrepancy may be attributed to three factors. First, rather than simply tracking regional macroclimatic shifts, these species are likely constrained by the Altai Mountain range, which extends in a northwest‐southeast direction, and may constrain species migration. Second, the climate warming in eastern mountainous regions is expected to be milder, thus posing a minor threat to rare and endangered plants (Figure S5) (Sanczuk et al. 2025). Third, this study focuses exclusively on rare and endangered plant species in the Irtysh River Basin rather than on all plant taxa. Such differences in research scope and target groups may also contribute to the divergent migration patterns observed.

### 4.2 The Unique Role of Mountain Regions

This study demonstrated that mountainous areas consistently played a pivotal role as suitable habitats and biodiversity hotspots for rare and endangered plant species under current and future climate and land use change scenarios (Figures 5 and 7). Specifically, species in mountainous and non‐mountainous areas were anticipated to exhibit distinct responses to climate change. Non‐mountainous species were predicted to undergo a significant habitat contraction, while mountainous species were predicted to undergo a habitat expansion. This disparity is primarily attributed to the complex topographic heterogeneity inherent to mountainous areas. The sharp elevation gradients and varying aspects and slopes of mountains create a rich array of microclimatic habitats on a small spatial scale (Scherrer and Körner 2011). These microclimatic habitats act as important refuges, offering great ecological niche space to plant species under climate change (Brighenti et al. 2021; Rahbek et al. 2019).

Furthermore, spatial disparities in anthropogenic activity intensity further bolster the role of mountainous areas as refugia for biodiversity. Plains and low‐elevation areas are subjected to high‐intensity anthropogenic disturbance from urbanization, agricultural expansion, and livestock husbandry. These activities have precipitated native vegetation loss and habitat fragmentation, compelling rare and endangered species to migrate toward mountainous areas to avoid anthropogenic pressures.

In this study, plant species were concentrated along mountainous rivers across the basin. This result aligns with the fact that flora in arid ecosystems predominantly colonize riparian zones to access water resources, and that mountainous areas in arid regions receive a relatively large amount of precipitation (Figure S6) (He et al. 2025; Viviroli et al. 2007). Therefore, despite the global trend of biodiversity loss, mountainous riparian zones are projected to consistently maintain a relatively high plant species richness and phylogenetic diversity due to their unique hydrothermal conditions.

### 4.3 Conservation of Rare and Endangered Plant Species

As the dual pressures of climate change and human activities intensify, the identification of Priority Conservation Areas (PCAs) has become a critical aspect of biodiversity conservation (Huang et al. 2016; Li et al. 2021; Myers et al. 2000). Previous studies have often relied solely on species distribution models (SDMs) to delineate conservation boundaries, and overlooked the actual limitations imposed by land use and land cover (LULC) conditions on species survival (Huang et al. 2020; Liu et al. 2025). This oversight can lead to an overprediction of suitable habitats. In this study, we integrated species distribution models with the InVEST habitat quality model to exclude areas that, despite being climatically suitable, have a low habitat quality due to anthropogenic pressures. This coupled approach not only enhances the accuracy of hotspot identification but also provides a reliable basis for developing feasible conservation plans (Huang et al. 2020; Wang et al. 2024).

Our findings revealed that the current conservation networks in the Irtysh River Basin had significant conservation gaps and spatial mismatches. Although these protected areas accounted for 12.87% of the study area, they had a severely insufficient ability to conserve rare and endangered plant species. Notably, approximately 81.25% of the studied species were classified as gap species (with protection coverage < 20%), indicating that the current protected area network has failed to effectively protect plant biodiversity. More critically, priority conservation areas only shared a 4.24% overlap with existing protected areas, and future biodiversity hotspots were expected to experience pronounced eastward and upward shifts. It should also be noted that some projected diversity‐gain areas may already be influenced by local conservation measures, including riparian forest enclosure and ecological restoration programs, which were not fully represented in the national/provincial protected‐area dataset used in this study. This limitation should be considered when interpreting conservation gaps in such areas. Nevertheless, the overall results suggest that the current protected‐area network may still be insufficient to accommodate the future redistribution of rare and endangered plants without timely adjustment.

To effectively address this issue, we propose the following conservation measures. First, we recommend optimizing the conservation network to address existing gaps. In the upper reaches of the Irtysh River, priority should be given to expanding existing protected areas and implementing flexible area‐based conservation measures. Because some of these areas are also used for grazing and other economic activities, zoned management and ecological compensation may offer more feasible and balanced options. Additionally, areas expected to experience significant increases in biodiversity should have their protection status elevated. Second, we recommend a tiered conservation approach. For species partly or entirely unprotected, in situ conservation should be implemented alongside enhanced monitoring of population dynamics. For species at high risk of future habitat loss, a combination of ex‐situ conservation and gene bank initiatives should be adopted. Third, we recommend establishing riparian ecological corridors. Our results reveal that hotspots for rare and endangered plant species are potentially impacted by human disturbances, so we advocate for strictly delineating ecological red lines and reinforcing management of human activity.

### 4.4 Limitations of the Study

To improve predictive reliability, we used an ensemble model approach, but several limitations remain. First, the 1 km resolution of the available environmental layers may not adequately capture fine‐scale heterogeneity in soil moisture, slope position, and riparian ecotones, all of which can strongly influence the persistence of rare and endangered plants in arid basins. As a result, some microrefugia may be overlooked, and the priority areas identified here should be interpreted as broad‐scale conservation priorities rather than precise site‐level targets. Second, species excluded because of very limited occurrence records may be among the most vulnerable taxa in the Irtysh River Basin. Because predictive modeling is inherently constrained by such data sparsity, their exclusion reflects an important source of uncertainty and highlights the need for targeted field surveys and long‐term population monitoring in future assessments. Third, we did not conduct additional species‐specific tuning of MaxEnt parameters, such as the regularization multiplier and feature classes, which may have affected model complexity and increased the risk of overfitting or underfitting. Consequently, uncertainty in species‐level suitability predictions and threshold‐based binary maps may propagate to multispecies stacking outputs, including SR, PD, hotspot identification, and conservation gap assessment. These effects should therefore be considered when interpreting fine‐scale spatial boundaries, especially around the margins of predicted suitable habitats, hotspots, and conservation gaps. Fourth, our modeling framework does not explicitly account for biotic interactions, such as interspecific competition or density‐dependent effects. Therefore, projected diversity‐gain areas should be interpreted as potential refugia rather than guaranteed sites of long‐term coexistence.

## 5 Conclusions

This study projected the spatial distribution of rare and endangered plants in the Irtysh River Basin, evaluated their differential responses to climatic alterations, and identified conservation gaps in current protected area networks. The results indicated that rare and endangered plants were concentrated along river banks, especially in mountainous riparian zones. Although most species were expected to undergo range contractions under future climate scenarios, leading to an overall decline in species richness and phylogenetic diversity, biodiversity in mountainous areas was projected to increase. This finding suggested that mountainous areas functioned as critical climate refugia, effectively buffering against the adverse impacts of environmental perturbations. As a result, it is imperative and urgent to intensify conservation efforts in these areas. This study provides important insights into the distribution patterns of rare and endangered plants in dryland regions, and underscores the critical importance of incorporating climate change into conservation planning to safeguard fragile ecosystems.

## Author Contributions

**Xun Lei:** investigation (equal), methodology (lead), software (equal), visualization (lead), writing – original draft (lead). **Mengjun Qu:** data curation (equal), investigation (equal). **Jianming Wang:** data curation (equal), investigation (equal), methodology (equal). **Yin Wang:** data curation (equal), investigation (equal). **Wenkai Wang:** investigation (equal). **Zhaoyang Wang:** investigation (equal). **Baocheng Guo:** methodology (equal). **Jingwen Li:** data curation (equal), funding acquisition (lead), investigation (equal), methodology (equal).

## Funding

This work was supported by National Natural Science Foundation of China, 32471705. The Third Xinjiang Scientific Expedition Program, 2021xjkk0600.

## Conflicts of Interest

The authors declare no conflicts of interest.

## Supporting information


**Appendix S1:** Figures and tables.
**Table S1:** 63 key protected plant species included in this study, their protection level and threat category on the National Key Protected Wild Plants (NKPWP) List, the Xinjiang Uygur Autonomous Region Key Protected Wild Plants (XKPWP) List, and the IUCN Red List of Threatened Species, and the number of occurrence records for each species.
**Table S2:** Threat factors and their maximum influence distance, weight, and spatial attenuation type.
**Table S3:** Habitat suitability of different land use types and sensitivity to threat sources.
**Table S4:** List of nature reserves in Xinjiang used in this study.
**Table S5:** Protection coverage (%) for rare and endangered species in current protected areas.
**Figure S1:** The true skill statistics (TSS) of species distribution ensemble models for rare and endangered plants in the Irtysh River Basin. Higher values indicate a better predictive performance of the model. The number of species within each interval of TSS is presented on the top of each bar.
**Figure S2:** Projected changes in suitable habitat areas for rare and endangered plants in mountainous and non‐mountainous areas.
**Figure S3:** Geographic centroid shift of climate variables under future climate scenarios.
**Figure S4:** Spatial distribution of habitat quality in the Irtysh River Basin under current and future scenarios. (a) Current; (b) Future.
**Figure S5:** The changes in climatic variables across the study area by 2050.
**Figure S6:** Current and future (2050) climatic conditions in the study area. (a) current; (b) future.
**Appendix S2:** Future land‐use simulation based on PLUS model.
**Table A1** The land‐use demand in 2050 (grids number) and neighborhood weights for each land‐use type.
**Table A2** The land‐use transition matrix in 2050.

## Data Availability

All the required data are uploaded as Supporting Information.

## References

[ece373769-bib-0001] Ackerly, D. D. , S. R. Loarie , W. K. Cornwell , et al. 2010. “The Geography of Climate Change: Implications for Conservation Biogeography.” Diversity and Distributions 16: 476–487. 10.1111/j.1472-4642.2010.00654.x.

[ece373769-bib-0002] Allouche, O. , A. Tsoar , and R. Kadmon . 2006. “Assessing the Accuracy of Species Distribution Models: Prevalence, Kappa and the True Skill Statistic (TSS).” Journal of Applied Ecology 43: 1223–1232. 10.1111/j.1365-2664.2006.01214.x.

[ece373769-bib-0003] Araújo, M. B. , and M. New . 2007. “Ensemble Forecasting of Species Distributions.” Trends in Ecology & Evolution 22: 42–47. 10.1016/j.tree.2006.09.010. 17011070

[ece373769-bib-0004] Barbet‐Massin, M. , F. Jiguet , C. H. Albert , and W. Thuiller . 2012. “Selecting Pseudo‐Absences for Species Distribution Models: How, Where and How Many?” Methods in Ecology and Evolution 3: 327–338. 10.1111/j.2041-210X.2011.00172.x.

[ece373769-bib-0005] Breiner, F. T. , A. Guisan , A. Bergamini , and M. P. Nobis . 2015. “Overcoming Limitations of Modelling Rare Species by Using Ensembles of Small Models.” Methods in Ecology and Evolution 6: 1210–1218. 10.1111/2041-210X.12403.

[ece373769-bib-0006] Brighenti, S. , S. Hotaling , D. S. Finn , et al. 2021. “Rock Glaciers and Related Cold Rocky Landforms: Overlooked Climate Refugia for Mountain Biodiversity.” Global Change Biology 27: 1504–1517. 10.1111/gcb.15510. 33404095

[ece373769-bib-0007] Chan, W.‐P. , J. Lenoir , G.‐S. Mai , H.‐C. Kuo , I.‐C. Chen , and S.‐F. Shen . 2024. “Climate Velocities and Species Tracking in Global Mountain Regions.” Nature 629: 114–120. 10.1038/s41586-024-07264-9. 38538797 PMC11062926

[ece373769-bib-0008] Chen, I.‐C. , J. K. Hill , R. Ohlemüller , D. B. Roy , and C. D. Thomas . 2011. “Rapid Range Shifts of Species Associated With High Levels of Climate Warming.” Sciences 333: 1024–1026. 10.1126/science.1206432. 21852500

[ece373769-bib-0009] Chen, Y.‐H. , J. Lenoir , and I.‐C. Chen . 2025. “Limited Evidence for Range Shift–Driven Extinction in Mountain Biota.” Sciences 388: 741–747. 10.1126/science.adq9512. 40373134

[ece373769-bib-0010] Dee, L. E. , J. Cowles , F. Isbell , S. Pau , S. D. Gaines , and P. B. Reich . 2019. “When Do Ecosystem Services Depend on Rare Species?” Trends in Ecology & Evolution 34: 746–758. 10.1016/j.tree.2019.03.010. 31104954

[ece373769-bib-0011] Ding, W.‐N. , R. H. Ree , R. A. Spicer , and Y.‐W. Xing . 2020. “Ancient Orogenic and Monsoon‐Driven Assembly of the World's Richest Temperate Alpine Flora.” Sciences 369: 578–581. 10.1126/science.abb4484. 32732426

[ece373769-bib-0012] Dolezal, J. , V. Jandova , M. Macek , et al. 2021. “Climate Warming Drives Himalayan Alpine Plant Growth and Recruitment Dynamics.” Journal of Ecology 109: 179–190. 10.1111/1365-2745.13459.

[ece373769-bib-0013] Dong, X. , J. Gong , X. Li , et al. 2024. “Effects of Future Climate Change on Rare and Endangered Species in Inner Mongolia, China: Vulnerability, Priority Conservation Areas and Sustainable Conservation Strategies.” Biodiversity and Conservation 33: 1961–1983. 10.1007/s10531-024-02830-z.

[ece373769-bib-0014] Elith, J. , and J. R. Leathwick . 2009. “Species Distribution Models: Ecological Explanation and Prediction Across Space and Time.” Annual Review of Ecology, Evolution, and Systematics 40: 677–697. 10.1146/annurev.ecolsys.110308.120159.

[ece373769-bib-0015] Elsen, P. R. , and M. W. Tingley . 2015. “Global Mountain Topography and the Fate of Montane Species Under Climate Change.” Nature Climate Change 5: 772–776. 10.1038/nclimate2656.

[ece373769-bib-0016] Etard, A. , A. L. Pigot , and T. Newbold . 2022. “Intensive Human Land Uses Negatively Affect Vertebrate Functional Diversity.” Ecology Letters 25: 330–343. 10.1111/ele.13926. 34816566

[ece373769-bib-0017] Faith, D. P. 1992. “Conservation Evaluation and Phylogenetic Diversity.” Biological Conservation 61: 1–10. 10.1016/0006-3207(92)91201-3.

[ece373769-bib-0018] Faith, D. P. 2013. “Biodiversity and Evolutionary History: Useful Extensions of the PD Phylogenetic Diversity Assessment Framework.” Annals of the New York Academy of Sciences 1289: 69–89. 10.1111/nyas.12186. 23773093

[ece373769-bib-0019] Faith, D. P. 2021. “A Singular Concept of Biodiversity Remains the Best Way to Address the Plural Values of Nature in Conservation Planning.” Conservation 1: 342–349. 10.3390/conservation1040026.

[ece373769-bib-0020] Gallardo, B. , D. C. Aldridge , P. González‐Moreno , et al. 2017. “Protected Areas Offer Refuge From Invasive Species Spreading Under Climate Change.” Global Change Biology 23: 5331–5343. 10.1111/gcb.13798. 28758293

[ece373769-bib-0021] Gotelli, N. J. , and R. K. Colwell . 2001. “Quantifying Biodiversity: Procedures and Pitfalls in the Measurement and Comparison of Species Richness.” Ecology Letters 4: 379–391. 10.1046/j.1461-0248.2001.00230.x.

[ece373769-bib-0022] Guo, Y. , Z. Zhao , S. Yuan , and X. Li . 2023. “A Greener Loess Plateau in the Future: Moderate Warming Will Expand the Potential Distribution Areas of Woody Species.” Environmental Research Letters 18: 34027. 10.1088/1748-9326/acb9a8.

[ece373769-bib-0023] He, B. , J. Chang , A. Guo , et al. 2025. “Spatial and Temporal Runoff Variability in Response to Climate Change in Alpine Mountains.” Journal of Hydrology 654: 132779. 10.1016/j.jhydrol.2025.132779.

[ece373769-bib-0024] Hijmans, R. J. 2024. “geosphere: Spherical Trigonometry.” CRAN.R‐project.org/package=geosphere.

[ece373769-bib-0025] Hijmans, R. J. 2025a. “raster: Geographic Data Analysis and Modeling.” CRAN.R‐project.org/package=raster.

[ece373769-bib-0026] Hijmans, R. J. 2025b. “terra: Spatial Data Analysis.” CRAN.R‐project.org/package=terra.

[ece373769-bib-0027] Hu, J.‐L. , X.‐Q. Ci , X.‐Y. Zhang , et al. 2024. “Assessment of Multidimensional Diversity and Conservation of Threatened Timber Trees in China Under Climate Change.” Biological Conservation 300: 110871. 10.1016/j.biocon.2024.110871.

[ece373769-bib-0028] Huang, J. , J. Huang , C. Liu , J. Zhang , X. Lu , and K. Ma . 2016. “Diversity Hotspots and Conservation Gaps for the Chinese Endemic Seed Flora.” Biological Conservation 198: 104–112. 10.1016/j.biocon.2016.04.007.

[ece373769-bib-0029] Huang, Z. , Y. Bai , J. M. Alatalo , and Z. Yang . 2020. “Mapping Biodiversity Conservation Priorities for Protected Areas: A Case Study in Xishuangbanna Tropical Area, China.” Biological Conservation 249: 108741. 10.1016/j.biocon.2020.108741.

[ece373769-bib-0030] Jin, Y. , and H. Qian . 2019. “V.PhyloMaker: An R Package That Can Generate Very Large Phylogenies for Vascular Plants.” Ecography 42: 1353–1359. 10.1111/ecog.04434. PMC9363651 35967255

[ece373769-bib-0031] Kembel, S. W. , P. D. Cowan , M. R. Helmus , et al. 2010. “Picante: R Tools for Integrating Phylogenies and Ecology.” Bioinformatics 26: 1463–1464. 10.1093/bioinformatics/btq166. 20395285

[ece373769-bib-0032] Keppel, G. , K. P. Van Niel , G. W. Wardell‐Johnson , et al. 2012. “Refugia: Identifying and Understanding Safe Havens for Biodiversity Under Climate Change.” Global Ecology and Biogeography 21: 393–404. 10.1111/j.1466-8238.2011.00686.x.

[ece373769-bib-0033] Li, G. , N. Xiao , Z. Luo , et al. 2021. “Identifying Conservation Priority Areas for Gymnosperm Species Under Climate Changes in China.” Biological Conservation 253: 108914. 10.1016/j.biocon.2020.108914.

[ece373769-bib-0034] Lian, X. , S. Piao , A. Chen , et al. 2021. “Multifaceted Characteristics of Dryland Aridity Changes in a Warming World.” Nature Reviews Earth and Environment 2: 232–250. 10.1038/s43017-021-00144-0.

[ece373769-bib-0035] Liu, F.‐L. , W. W. Mambo , J. Liu , et al. 2025. “Spatiotemporal Range Dynamics and Conservation Optimization for Endangered Medicinal Plants in the Himalaya.” Global Ecology and Conservation 57: e03390. 10.1016/j.gecco.2024.e03390.

[ece373769-bib-0036] Liu, H. , J. Fan , B. Liu , L. Wang , and Q. Qiao . 2021. “Practical Exploration of Ecological Restoration and Management of the Mountains‐Rivers‐Forests‐Farmlands‐Lakes‐Grasslands System in the Irtysh River Basin in Altay, Xinjiang.” Journal of Resources and Ecology 12: 766–776. 10.5814/j.issn.1674-764x.2021.06.005.

[ece373769-bib-0037] Liu, H. , H. Hao , W. Zhang , P. Liu , and L. Sun . 2022. “Evaluation of Ecological Protection and Restoration Effectiveness Based On ‘Pattern‐Quality‐Service’ in Irtysh River Basin.” Research of Environmental Sciences 35: 2495–2507. 10.13198/j.issn.1001-6929.2022.09.29.

[ece373769-bib-0038] Lu, M. , H. Wang , and X. Xun . 2024. “Spatial‐Temporal Evolution of Habitat Quality in Altay Area and Its Driving Factors.” Journal of Beijing Forestry University 46: 27–39. 10.12171/j.1000-1522.20230189.

[ece373769-bib-0039] Luo, W. , H. Sun , F. Zhong , et al. 2022. “Effects of Land Use Change on Spatio‐Temporal Evolution of Ecosystem Service Value Profit and Loss in Irtysh River Basin.” Bulletin of Soil and Water Conservation 42: 301–311. 10.13961/j.cnki.stbctb.2022.04.038.

[ece373769-bib-0040] Mahmoodi, S. , K. Ahmadi , M. Heydari , O. Karami , O. Esmailzadeh , and B. Heung . 2023. “Elevational Shift of Endangered European Yew Under Climate Change in Hyrcanian Mountain Forests: Rethinking Conservation‐Restoration Strategies and Management.” Forest Ecology and Management 529: 120693. 10.1016/j.foreco.2022.120693.

[ece373769-bib-0041] Marmion, M. , M. Parviainen , M. Luoto , R. K. Heikkinen , and W. Thuiller . 2009. “Evaluation of Consensus Methods in Predictive Species Distribution Modelling.” Diversity and Distributions 15: 59–69. 10.1111/j.1472-4642.2008.00491.x.

[ece373769-bib-0042] Men, D. , and J. Pan . 2025. “Integrating Key Species Distribution and Ecosystem Service Flows to Build Directed Ecological Network: Evidence From the Shiyang River Basin, China.” Journal of Environmental Management 381: 125183. 10.1016/j.jenvman.2025.125183. 40199207

[ece373769-bib-0043] Myers, N. , R. A. Mittermeier , C. G. Mittermeier , G. A. B. da Fonseca , and J. Kent . 2000. “Biodiversity Hotspots for Conservation Priorities.” Nature 403: 853–858. 10.1038/35002501. 10706275

[ece373769-bib-0044] Natural Capital Project . 2022. InVEST 3.13.0 User's Guide. Stanford University, University of Minnesota, Chinese Academy of Sciences, The Nature Conservancy, World Wildlife Fund, and Stockholm Resilience Centre.

[ece373769-bib-0045] Newbold, T. , L. N. Hudson , A. P. Arnell , et al. 2016. “Has Land Use Pushed Terrestrial Biodiversity Beyond the Planetary Boundary? A Global Assessment.” Sciences 353: 288–291. 10.1126/science.aaf2201. 27418509

[ece373769-bib-0046] Orme, C. D. L. , R. G. Davies , M. Burgess , et al. 2005. “Global Hotspots of Species Richness Are Not Congruent With Endemism or Threat.” Nature 436: 1016–1019. 10.1038/nature03850. 16107848

[ece373769-bib-0047] Owen, N. R. , R. Gumbs , C. L. Gray , and D. P. Faith . 2019. “Global Conservation of Phylogenetic Diversity Captures More Than Just Functional Diversity.” Nature Communications 10: 859. 10.1038/s41467-019-08600-8. PMC6382770 30787282

[ece373769-bib-0048] Pearson, R. G. , C. J. Raxworthy , M. Nakamura , and A. Townsend Peterson . 2007. “ORIGINAL ARTICLE: Predicting Species Distributions From Small Numbers of Occurrence Records: A Test Case Using Cryptic Geckos in Madagascar.” Journal of Biogeography 34: 102–117. 10.1111/j.1365-2699.2006.01594.x.

[ece373769-bib-0049] Peterson, A. T. , M. Papeş , and J. Soberón . 2008. “Rethinking Receiver Operating Characteristic Analysis Applications in Ecological Niche Modeling.” Ecological Modelling 213: 63–72. 10.1016/j.ecolmodel.2007.11.008.

[ece373769-bib-0050] Pillet, M. , B. Goettsch , C. Merow , et al. 2022. “Elevated Extinction Risk of Cacti Under Climate Change.” Nature Plants 8: 366–372. 10.1038/s41477-022-01130-0. 35422081

[ece373769-bib-0051] Pinkert, S. , N. Farwig , A. Y. Kawahara , and W. Jetz . 2025. “Global Hotspots of Butterfly Diversity Are Threatened in a Warming World.” Nature Ecology & Evolution 9: 1–12. 10.1038/s41559-025-02664-0. 40128452 PMC12066357

[ece373769-bib-0052] Rahbek, C. , M. K. Borregaard , R. K. Colwell , et al. 2019. “Humboldt's Enigma: What Causes Global Patterns of Mountain Biodiversity?” Sciences 365: 1108–1113. 10.1126/science.aax0149. 31515383

[ece373769-bib-0053] Salmerón Gómez, R. , J. García Pérez , M. D. M. López Martín , and C. G. García . 2016. “Collinearity Diagnostic Applied in Ridge Estimation Through the Variance Inflation Factor.” Journal of Applied Statistics 43: 1831–1849. 10.1080/02664763.2015.1120712.

[ece373769-bib-0054] Sanczuk, P. , J. Lenoir , P. Denelle , et al. 2025. “Global Bias Towards Recording Latitudinal Range Shifts.” Nature Climate Change 16: 1–5. 10.1038/s41558-025-02498-5.

[ece373769-bib-0055] Scherrer, D. , and C. Körner . 2011. “Topographically Controlled Thermal‐Habitat Differentiation Buffers Alpine Plant Diversity Against Climate Warming.” Journal of Biogeography 38: 406–416. 10.1111/j.1365-2699.2010.02407.x.

[ece373769-bib-0056] Schwartz, M. W. , L. R. Iverson , A. M. Prasad , S. N. Matthews , and R. J. O'Connor . 2006. “Predicting Extinctions as a Result of Climate Change.” Ecology 87: 1611–1615. 10.1890/0012-9658(2006)87[1611:PEAARO]2.0.CO;2. 16922312

[ece373769-bib-0057] Song, H. , A. Ordonez , J.‐C. Svenning , et al. 2021. “Regional Disparity in Extinction Risk: Comparison of Disjunct Plant Genera Between Eastern Asia and Eastern North America.” Global Change Biology 27: 1904–1914. 10.1111/gcb.15525. 33474767

[ece373769-bib-0058] Sun, Y. , Y. Deng , S. Yao , et al. 2025. “Distribution Range and Richness of Plant Species Are Predicted to Increase by 2100 due to a Warmer and Wetter Climate in Northern China.” Global Change Biology 31: e70334. 10.1111/gcb.70334. 40613311 PMC12232221

[ece373769-bib-0059] Thuiller, W. , B. Lafourcade , R. Engler , and M. B. Araújo . 2009. “BIOMOD—A Platform for Ensemble Forecasting of Species Distributions.” Ecography 32: 369–373. 10.1111/j.1600-0587.2008.05742.x.

[ece373769-bib-0060] Tucker, C. M. , M. W. Cadotte , S. B. Carvalho , et al. 2017. “A Guide to Phylogenetic Metrics for Conservation, Community Ecology and Macroecology.” Biological Reviews 92: 698–715. 10.1111/brv.12252. 26785932 PMC5096690

[ece373769-bib-0061] Vincent, H. , C. N. Bornand , A. Kempel , and M. Fischer . 2020. “Rare Species Perform Worse Than Widespread Species Under Changed Climate.” Biological Conservation 246: 108586. 10.1016/j.biocon.2020.108586.

[ece373769-bib-0062] Viviroli, D. , H. H. Dürr , B. Messerli , M. Meybeck , and R. Weingartner . 2007. “Mountains of the World, Water Towers for Humanity: Typology, Mapping, and Global Significance.” Water Resources Research 43: W07447. 10.1029/2006WR005653.

[ece373769-bib-0063] Voskamp, A. , S. A. Fritz , V. Köcke , et al. 2023. “Utilizing Multi‐Objective Decision Support Tools for Protected Area Selection.” One Earth 6: 1143–1156. 10.1016/j.oneear.2023.08.009.

[ece373769-bib-0064] Wang, B. , and W. Cheng . 2022. “Effects of Land Use/Cover on Regional Habitat Quality Under Different Geomorphic Types Based on InVEST Model.” Remote Sensing 14: 1279. 10.3390/rs14051279.

[ece373769-bib-0065] Wang, F. , X. Yuan , Y. Sun , and Y. Liu . 2024. “Species Distribution Modeling Based on MaxEnt to Inform Biodiversity Conservation in the Central Urban Area of Chongqing Municipality.” Ecological Indicators 158: 111491. 10.1016/j.ecolind.2023.111491.

[ece373769-bib-0066] Wang, S. , X. Wang , X. Han , and Y. Deng . 2018. “Higher Precipitation Strengthens the Microbial Interactions in Semi‐Arid Grassland Soils.” Global Ecology and Biogeography 27: 570–580. 10.1111/geb.12718.

[ece373769-bib-0067] Wang, X. , B. Guo , and L. Zhang . 2016. “Protection Values on Xinjiang Irtysh Keketuohai Wetland Nature Reserve.” Forest Resources Management 5: 6–12. 10.13466/j.cnki.lyzygl.2016.05.002.

[ece373769-bib-0068] Whittaker, R. H. 1972. “Evolution and Measurement of Species Diversity.” Taxon 21: 213–251. 10.2307/1218190.

[ece373769-bib-0069] Winter, M. , V. Devictor , and O. Schweiger . 2013. “Phylogenetic Diversity and Nature Conservation: Where Are We?” Trends in Ecology & Evolution 28: 199–204. 10.1016/j.tree.2012.10.015. 23218499

[ece373769-bib-0070] Wu, Y. , J. Shen , D. C. Deane , et al. 2025. “Future Extreme Climate Events Threaten Alpine and Subalpine Woody Plants in China.” Earth's Future 13: e2024EF005147. 10.1029/2024EF005147.

[ece373769-bib-0071] Xu, L. , T. Liu , Z. Xue , et al. 2024. “Unique Plant Resources and Distribution Patterns in the Valley Forest of the Irtysh River Basin.” Plants 13: 1957. 10.3390/plants13141957. 39065484 PMC11281289

[ece373769-bib-0072] Yang, H. , H. Zhang , Y. Wang , et al. 2025. “Urban Bird Diversity Conservation Plan Based on the MaxEnt Model and InVEST Model: A Case Study of Jinan, China.” Ecological Indicators 174: 113463. 10.1016/j.ecolind.2025.113463.

[ece373769-bib-0073] Ye, P. , G. Zhang , X. Zhao , H. Chen , Q. Si , and J. Wu . 2021. “Potential Geographical Distribution and Environmental Explanations of Rare and Endangered Plant Species Through Combined Modeling: A Case Study of Northwest Yunnan, China.” Ecology and Evolution 11: 13052–13067. 10.1002/ece3.7999. 34646452 PMC8495784

[ece373769-bib-0074] Zhang, J. , S. E. Nielsen , Y. Chen , et al. 2017. “Extinction Risk of North American Seed Plants Elevated by Climate and Land‐Use Change.” Journal of Applied Ecology 54: 303–312. 10.1111/1365-2664.12701.

[ece373769-bib-0075] Zhou, Q. , X. Li , Y. Wang , Z. Xin , A. Musa , and L. Wang . 2023. “Mesophytic and Less‐Disturbed Mountainous Habitats Are Important for In Situ Conservation of Rare and Endangered Plants.” Global Ecology and Conservation 44: e02488. 10.1016/j.gecco.2023.e02488.

[ece373769-bib-0076] Zhu, Y. , X. Xu , Z. Xi , and J. Liu . 2023. “Conservation Priorities for Endangered Trees Facing Multiple Threats Around the World.” Conservation Biology 37: e14142. 10.1111/cobi.14142. 37424365

