# Supplementary material for: Mountain Riparian Zones as Refugia for Rare and Endangered Plants Under Climate Change
Source: Ecol Evol. 2026 Jun 1;16(6):e73769. doi: 10.1002/ece3.73769 (PMC13239824; doi:10.1002/ece3.73769)
Supplement: Supplementary file 1 — Appendix S1: Figures and tables. Table S1: 63 key protected plant species included in this study, their protection level and threat category on the National Key Protected Wild Plants (NKPWP) List, the Xinjiang Uygur Autonomous Region Key Protected Wild Plants (XKPWP) List, and the IUCN Red List of Threatened Species, and the number of occurrence records for each species. Table S2: Threat factors and their maximum influence distance, weight, and spatial attenuation type. Table S3: Habitat suitability of different land use types and sensitivity to threat sources. Table S4: List of nature reserves in Xinjiang used in this study. Table S5: Protection coverage (%) for rare and endangered species in current protected areas. Figure S1: The true skill statistics (TSS) of species distribution ensemble models for rare and endangered plants in the Irtysh River Basin. Higher values indicate a better predictive performance of the model. The number of species within each interval of TSS is presented on the top of each bar. Figure S2: Projected changes in suitable habitat areas for rare and endangered plants in mountainous and non‐mountainous areas. Figure S3: Geographic centroid shift of climate variables under future climate scenarios. Figure S4: Spatial distribution of habitat quality in the Irtysh River Basin under current and future scenarios. (a) Current; (b) Future. Figure S5: The changes in climatic variables across the study area by 2050. Figure S6: Current and future (2050) climatic conditions in the study area. (a) current; (b) future. Appendix S2: Future land‐use simulation based on PLUS model. Table A1 The land‐use demand in 2050 (grids number) and neighborhood weights for each land‐use type. Table A2 The land‐use transition matrix in 2050. [file ECE3-16-e73769-s001.docx]

**Supplementary Information**

**Appendix S1.** Figures S1–S6 and Tables S1–S5.

**Appendix S2.** Future land-use simulation based on PLUS model

**Appendix S1. Figures and tables.**

**Table S1** 63 key protected plant species included in this study, their protection level and threat category on the National Key Protected Wild Plants (NKPWP) List, the Xinjiang Uygur Autonomous Region Key Protected Wild Plants (XKPWP) List , and the IUCN Red List of Threatened Species, and the number of occurrence records for each species.

| Species | NKPWP | XKPWP | IUCN | Total Records | Records for model |
| --- | --- | --- | --- | --- | --- |
| *Tulipa altaica* | Ⅱ |  |  | 7 | 0 |
| *Tulipa heteropetala* | Ⅱ |  |  | 45 | 36 |
| *Tulipa uniflora* | Ⅱ |  |  | 10 | 9 |
| *Tulipa biflora* | Ⅱ |  |  | 7 | 5 |
| *Tulipa sinkiangensis* | Ⅱ |  |  | 8 | 0 |
| *Agropyron mongolicum* | Ⅱ |  |  | 12 | 12 |
| *Cistanche deserticola* | Ⅱ |  |  | 5 | 0 |
| *Ferula sinkiangensis* | Ⅱ |  |  | 6 | 5 |
| *Glycyrrhiza uralensis* | Ⅱ |  |  | 131 | 91 |
| *Lycium ruthenicum* | Ⅱ |  |  | 30 | 12 |
| *Rhodiola quadrifida* | Ⅱ |  |  | 8 | 6 |
| *Rhodiola rosea* | Ⅱ |  |  | 14 | 7 |
| *Populus ×berolinensis var. jrtyschensis* | Ⅱ |  |  | 66 | 30 |
| *Prunus tenella* | Ⅱ |  |  | 16 | 8 |
| *Cynomorium songaricum* | Ⅱ |  |  | 8 | 7 |
| *Fritillaria verticillata* | Ⅱ |  |  | 1 | 0 |
| *Tulipa kolpakowskiana* | Ⅱ |  |  | 2 | 0 |
| *Tulipa × gesneriana* | Ⅱ |  |  | 3 | 0 |
| *Saussurea orgaadayi* | Ⅱ |  |  | 3 | 0 |
| *Nymphaea candida* | Ⅱ |  |  | 2 | 0 |
| *Malus sieversii* | Ⅱ |  | VU | 2 | 0 |
| *Paeonia anomala* |  | Ⅰ |  | 54 | 37 |
| *Paeonia intermedia* |  | Ⅰ |  | 64 | 40 |
| *Lilium martagon var. pilosiusculum* |  | Ⅰ |  | 29 | 21 |
| *Pinus sibirica* |  | Ⅰ |  | 18 | 14 |
| *Abies sibirica* |  | Ⅰ |  | 9 | 8 |
| *Epipogium aphyllum* |  | Ⅰ |  | 9 | 0 |
| *Haloxylon ammodendron* |  | Ⅰ |  | 51 | 17 |
| *Juniperus semiglobosa* |  | Ⅰ |  | 2 | 0 |
| *Eutrema salsugineum* |  | Ⅰ |  | 2 | 0 |
| *Polypodium vulgare* |  | Ⅰ |  | 3 | 0 |
| *Prangos didyma* |  | Ⅰ |  | 2 | 0 |
| *Rhaponticum carthamoides* |  | Ⅰ |  | 2 | 0 |
| *Juniperus sabina* |  | Ⅱ |  | 64 | 47 |
| *Leymus racemosus* |  | Ⅱ |  | 45 | 36 |
| *Juniperus communis var. saxatilis* |  | Ⅱ |  | 46 | 38 |
| *Viola altaica* |  | Ⅱ |  | 11 | 9 |
| *Juniperus pseudosabina* |  | Ⅱ |  | 16 | 13 |
| *Ephedra intermedia* |  | Ⅱ |  | 32 | 13 |
| *Ephedra equisetina* |  | Ⅱ |  | 12 | 8 |
| *Erythronium sibiricum* |  | Ⅱ |  | 36 | 20 |
| *Allium altaicum* |  | Ⅱ | NT | 21 | 16 |
| *Goodyera repens* |  | Ⅱ |  | 24 | 11 |
| *Dactylorhiza umbrosa* |  | Ⅱ |  | 21 | 18 |
| *Allium roborowskianum* |  | Ⅱ |  | 15 | 12 |
| *Spiranthes sinensis* |  | Ⅱ |  | 10 | 8 |
| *Vaccinium vitis-idaea* |  | Ⅱ |  | 6 | 6 |
| *Eremosparton songoricum* |  | Ⅱ |  | 8 | 0 |
| *Allium galanthum* |  | Ⅱ |  | 13 | 0 |
| *Allium decipiens* |  | Ⅱ |  | 10 | 0 |
| *Sorbus sibirica* |  | Ⅱ |  | 3 | 0 |
| *Daphne altaica* |  | Ⅱ |  | 2 | 0 |
| *Vaccinium myrtillus* |  | Ⅱ |  | 2 | 0 |
| *Nymphoides peltata* |  | Ⅱ |  | 3 | 0 |
| *Lycium dasystemum* |  | Ⅱ |  | 4 | 0 |
| *Allium obliquum* |  | Ⅱ |  | 2 | 0 |
| *Epipactis palustris* |  | Ⅱ |  | 3 | 0 |
| *Populus pruinosa* |  | Ⅱ |  | 2 | 0 |
| *Oreosalsola arbusculiformis* |  | Ⅱ |  | 3 | 0 |
| *Gymnocarpos przewalskii* |  | Ⅱ |  | 3 | 0 |
| *Crataegus songarica* |  | Ⅱ |  | 3 | 0 |
| *Prunus tianshanica* |  | Ⅱ |  | 4 | 0 |
| *Calophaca soongorica* |  | Ⅱ |  | 2 | 0 |
| *Picea asperata* |  |  | VU | 8 | 0 |
| *Gymnospermium microrrhynchum* |  |  | EN | 1 | 0 |
| *Populus pruinosa* |  |  | NT | 2 | 0 |
| Species Total Records |  |  |  | 1068 | 620 |

Note: Protection levels: Species with higher level (Ⅰ) indicates more endangered. IUCN threat category: Extinction (EX), Extinct in the wild (EW), Critically endangered (CR), Endangered (EN), Vulnerable (VU), Near threatened (NT), Least concern (LC), Data deficient (DD), Not evaluated (NE).

**Table S2** Threat factors and their maximum influence distance, weight, and spatial attenuation type

| Threat source type | Max. influencing distance/km | Weight | Spatial decay type |
| --- | --- | --- | --- |
| Cultivated land | 6.0 | 0.6 | Linear |
| Construction land | 8.0 | 0.8 | Exponential |
| Unused land | 5.0 | 0.7 | Linear |

**Table S3** Habitat suitability of different land use types and sensitivity to threat sources

| Land use type | Habitat suitability | Threat source | | |
| --- | --- | --- | --- | --- |
|  |  | Cultivated land | Construction land | Unused land |
| Cultivated land | 0.5 | 0 | 0.7 | 0.4 |
| Forestland | 1 | 0.7 | 0.85 | 0.5 |
| Grassland | 0.8 | 0.7 | 0.8 | 0.7 |
| Waters | 0.9 | 0.45 | 0.7 | 0.4 |
| Construction land | 0 | 0 | 0 | 0 |
| Unused land | 0.3 | 0.4 | 0.55 | 0 |

**Table S4** List of nature reserves in Xinjiang used in this study

|  | Name | Level |
| --- | --- | --- |
| 1 | Altai Mountains Two-River Source Nature Reserve | Provincial |
| 2 | Xinjiang Irtysh Keketuohai Wetland Nature Reserve | Provincial |
| 3 | Mountain Kalamaili Ungulate Nature Reserve | Provincial |
| 4 | Xinjiang Jintasi Mountain Grassland Provincial Nature Reserve | Provincial |
| 5 | Xinjiang Kanas National Nature Reserve | National |
| 6 | Xinjiang Altay Kekesu Wetland National Nature Reserve | National |
| 7 | Xinjiang Buergen Beaver National Nature Reserve | National |

**Table S5**. Protection coverage (%) for rare and endangered species in current protected areas.

|  | Species | Percentage of habitat area under protection (%) |
| --- | --- | --- |
| 1 | *Tulipa biflora* | 0.00 |
| 2 | *Lycium ruthenicum* | 2.21 |
| 3 | *Allium roborowskianum* | 2.76 |
| 4 | *Agropyron mongolicum* | 3.16 |
| 5 | *Haloxylon ammodendron* | 3.60 |
| 6 | *Erythronium sibiricum* | 3.71 |
| 7 | *Spiranthes sinensis* | 3.81 |
| 8 | *Tulipa heteropetala* | 4.36 |
| 9 | *Ephedra equisetina* | 5.34 |
| 10 | *Ephedra intermedia* | 6.15 |
| 11 | *Juniperus sabina* | 6.36 |
| 12 | *Abies sibirica* | 8.91 |
| 13 | *Cynomorium songaricum* | 9.64 |
| 14 | *Dactylorhiza umbrosa* | 10.50 |
| 15 | *Paeonia intermedia* | 10.71 |
| 16 | *Viola altaica* | 11.55 |
| 17 | *Glycyrrhiza uralensis* | 12.85 |
| 18 | *Juniperus communis var. saxatilis* | 13.52 |
| 19 | *Prunus tenella* | 14.32 |
| 20 | *Leymus racemosus* | 14.70 |
| 21 | *Paeonia anomala* | 14.75 |
| 22 | *Juniperus pseudosabina* | 15.23 |
| 23 | *Populus × berolinensis var. jrtyschensis* | 15.80 |
| 24 | *Ferula sinkiangensis* | 15.94 |
| 25 | *Vaccinium vitis-idaea* | 17.75 |
| 26 | *Tulipa uniflora* | 17.81 |
| 27 | *Pinus sibirica* | 18.73 |
| 28 | *Lilium martagon var. pilosiusculum* | 24.64 |
| 29 | *Rhodiola quadrifida* | 26.39 |
| 30 | *Goodyera repens* | 26.99 |
| 31 | *Rhodiola rosea* | 29.73 |
| 32 | *Allium altaicum* | 34.79 |


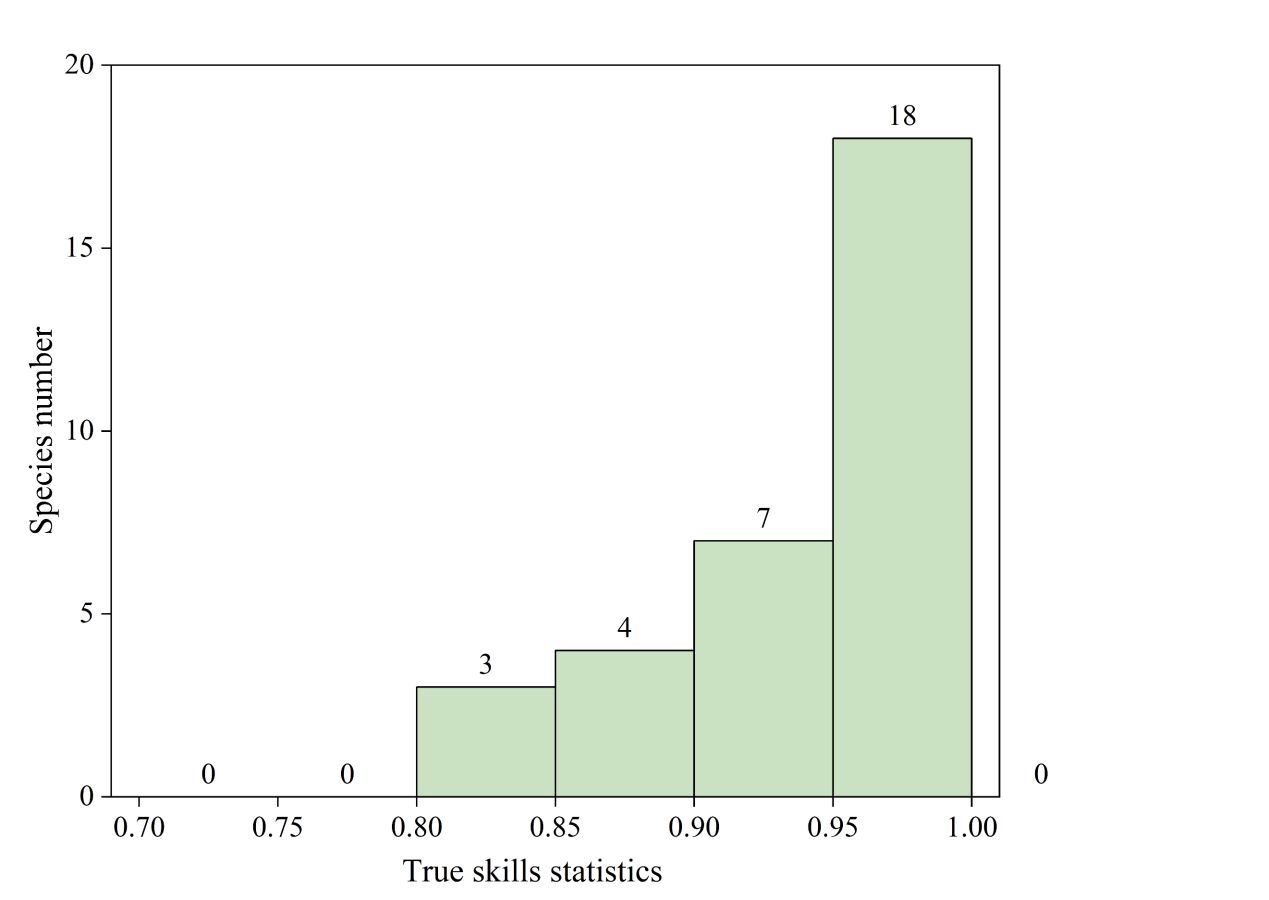


**Fig.S1** The true skill statistics (TSS) of species distribution ensemble models for rare and endangered plants in the Irtysh River Basin. Higher values indicate a better predictive performance of the model. The number of species within each interval of TSS is presented on the top of each bar.


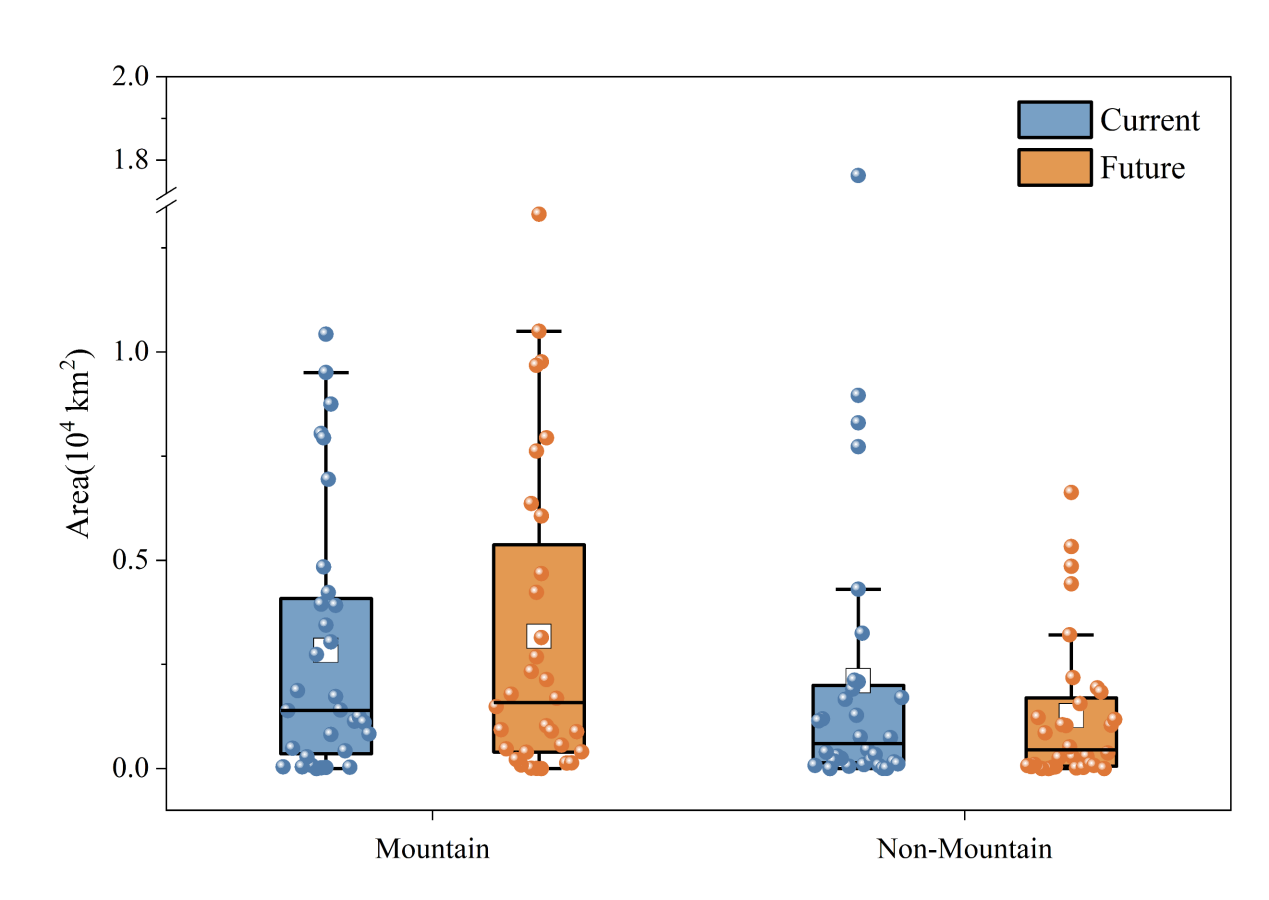


**Fig.S2** Projected changes in suitable habitat areas for rare and endangered plants in mountainous and non-mountainous areas


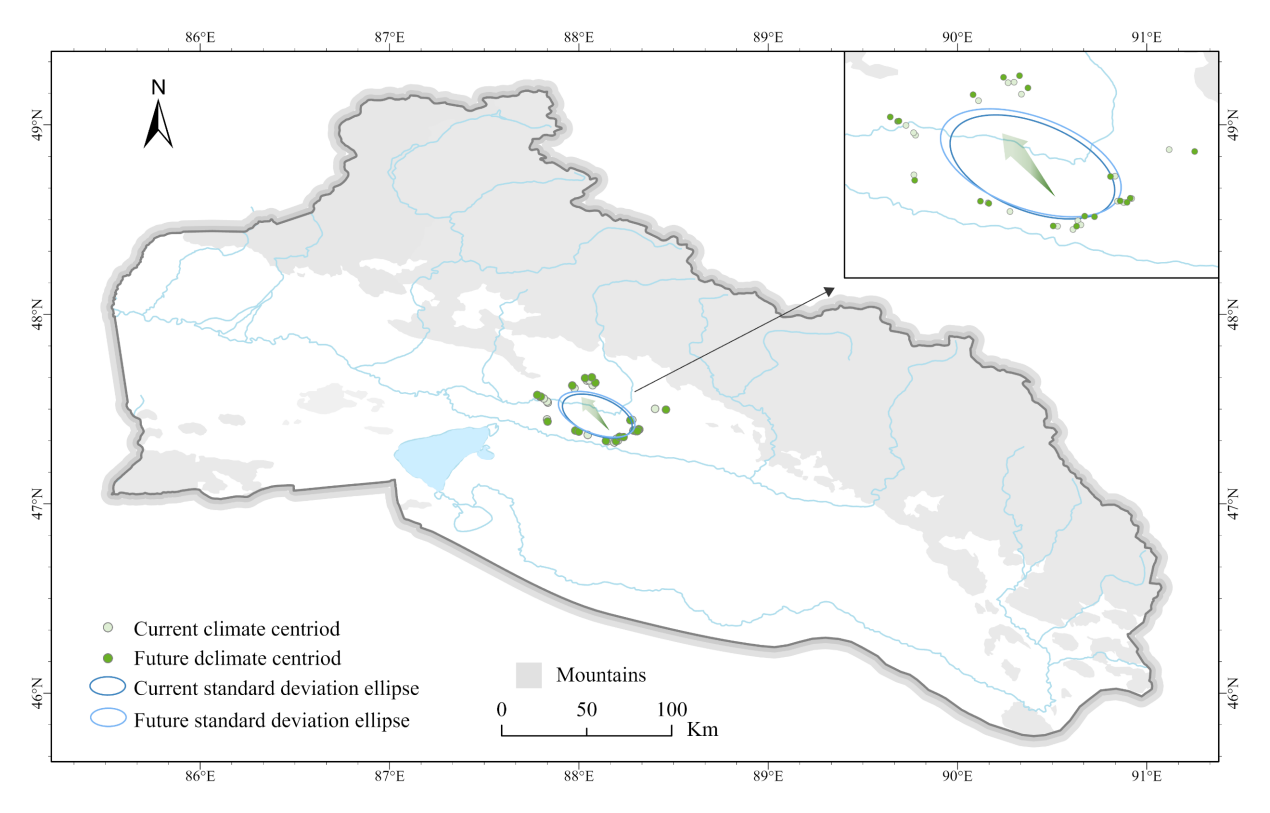


**Fig.S3** Geographic centroid shift of climate variables under future climate scenarios.


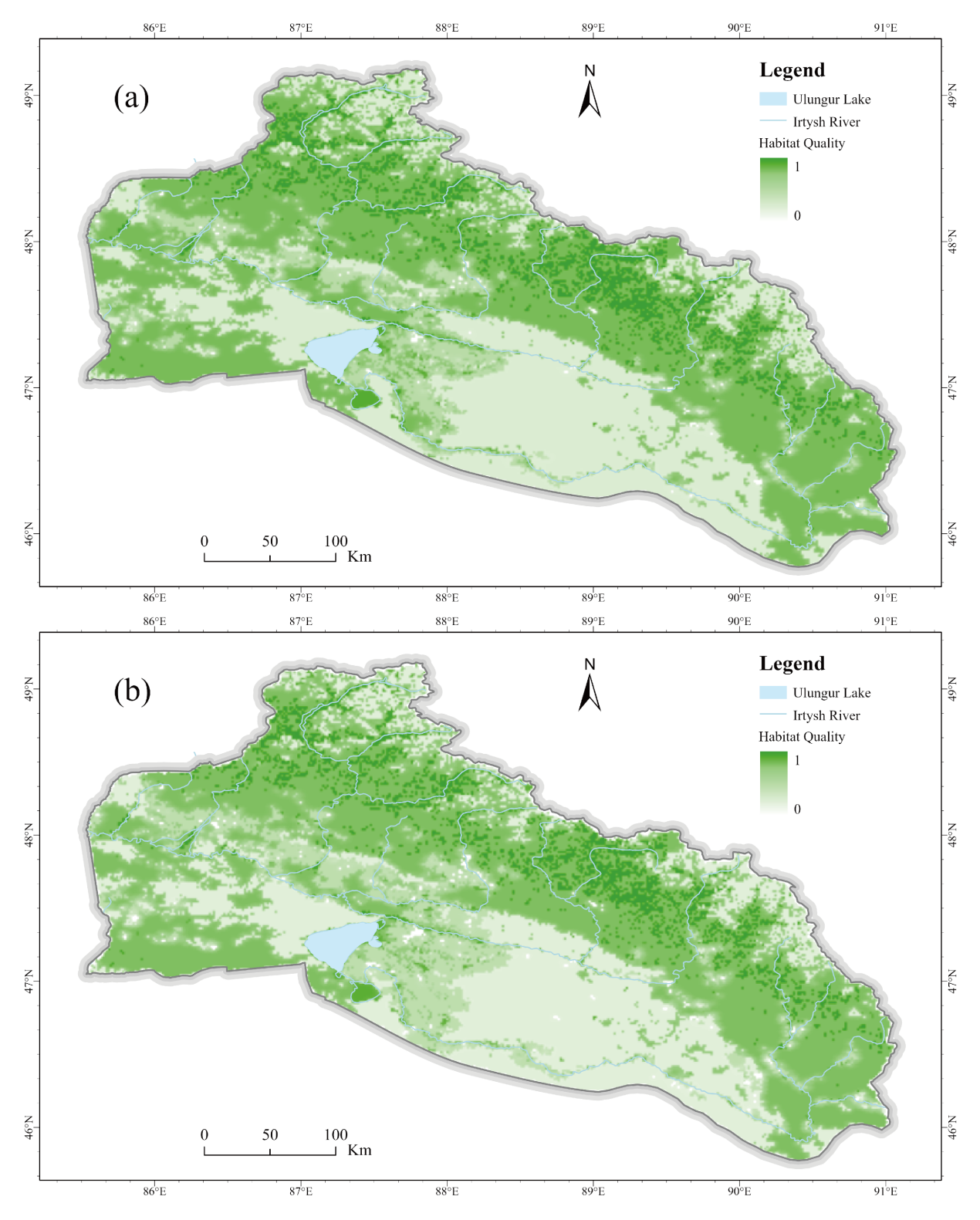


**Fig. S4.** Spatial distribution of habitat quality in the Irtysh River Basin under current and future scenarios. (a) Current; (b) Future.


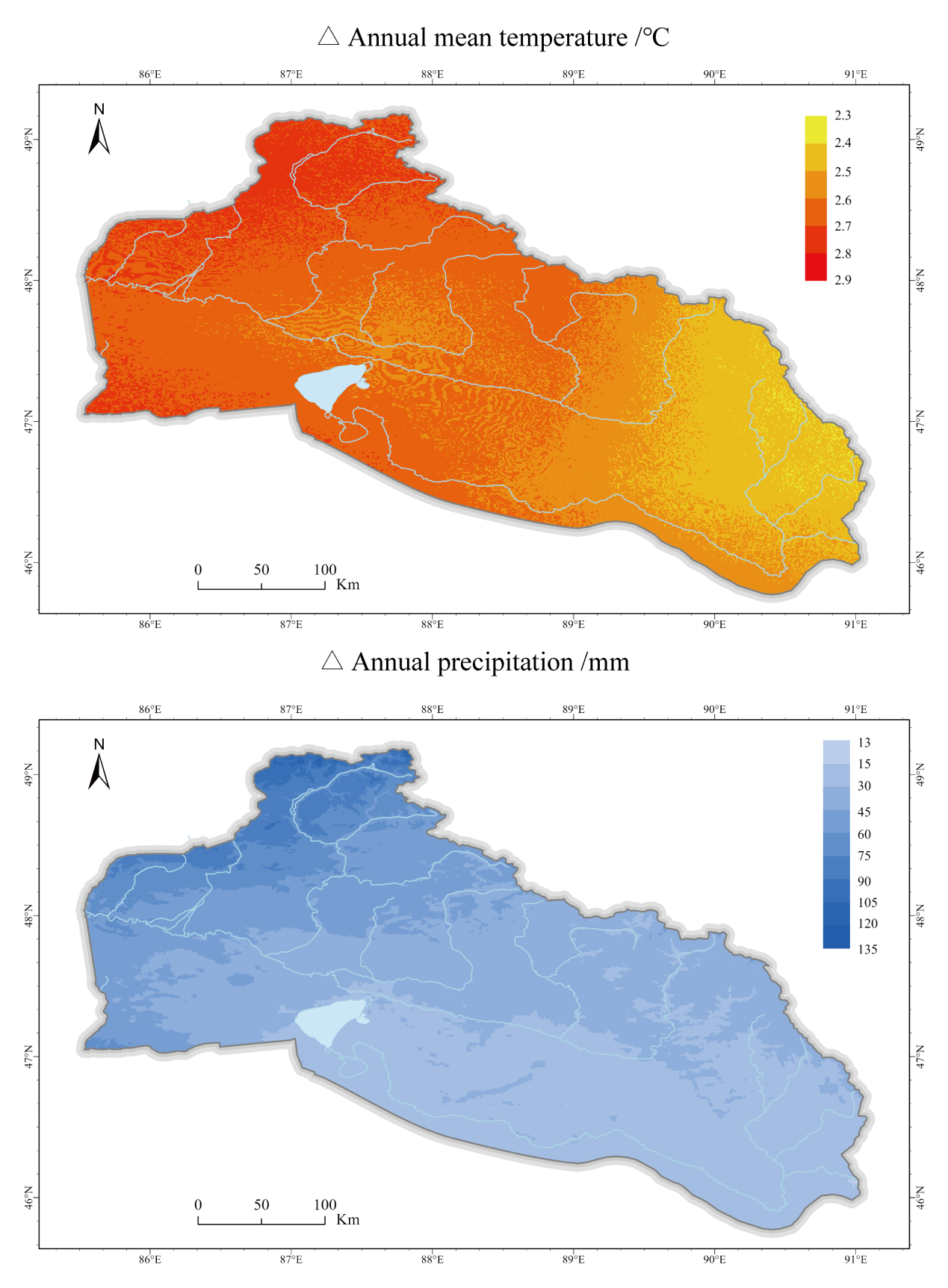


**Fig.S5** The changes in climatic variables across the study area by 2050.


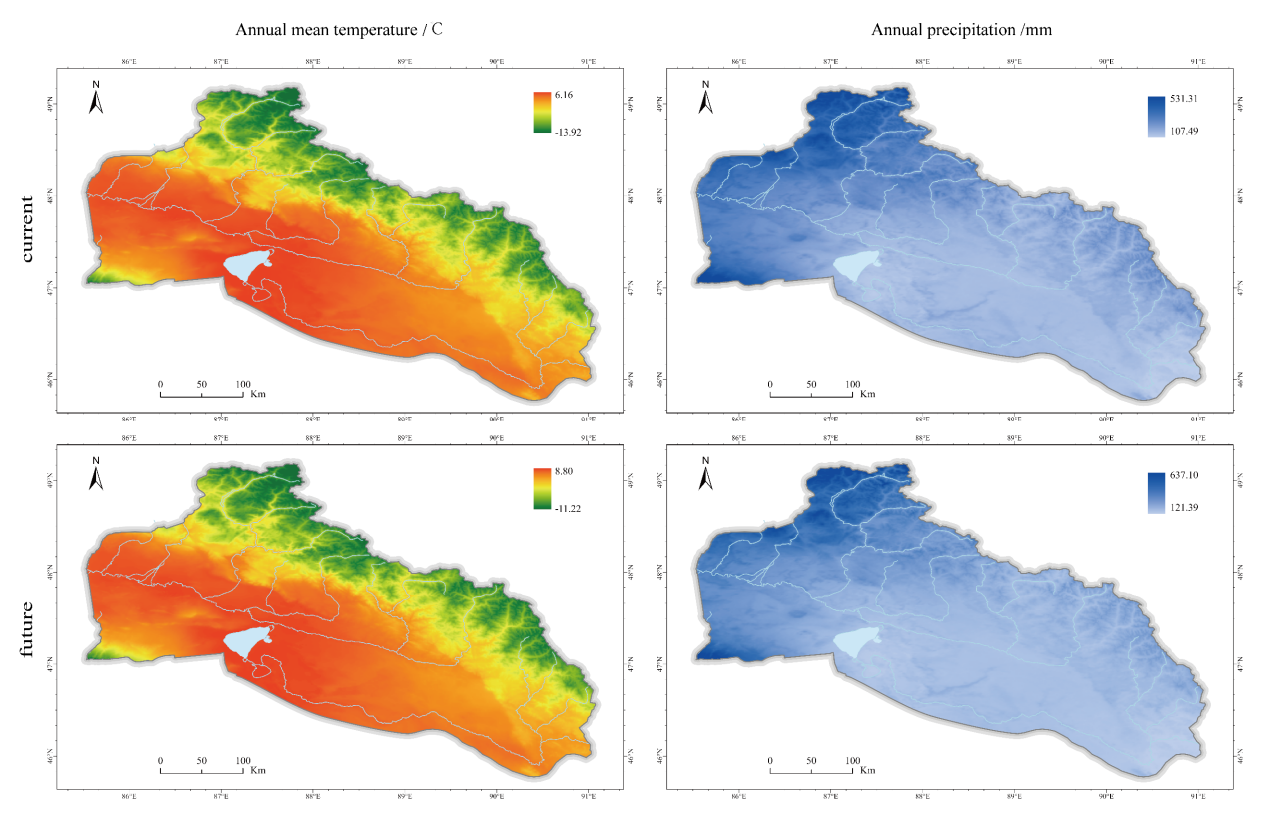


**Fig.S6** Current and future (2050) climatic conditions in the study area. (a) current; (b)future

**Appendix S2.** Future land-use simulation based on PLUS model

The PLUS model is principally constituted by two core modules: the land expansion analysis strategy (LEAS) and a cellular automata model based on multi-type random patch seeds (CARS)(Liang et al., 2021). Specifically, the LEAS module employs the random forest algorithm to elucidate the relationships between the expansion of various land-use categories derived from two distinct periods of land-use data and their corresponding driving factors. Consequently, it extracts the development probability for each land-use type alongside the contribution rates of the underlying drivers(Wang et al., 2022, 2023). Conversely, the CARS module integrates a random seed generation mechanism with a threshold decrement strategy to achieve the dynamic generation and simulation of patches across spatiotemporal dimensions, operating under the constraints of development probability(Xu et al., 2022).

In this study, the 2050 land-use projection was conducted under a Natural Development scenario (NDS), which assumes that historical land-use transition trends from 2000 to 2020 will continue through 2050 without significant policy interventions. Based on historical land-use data from 2000, 2010, and 2020, land-use demand in 2050 was projected using linear regression analysis (Table A1). The parameter configuration for the random forest algorithm within the LEAS module was established as follows: the number of regression trees was set to 20, the sampling rate to 0.01, mTry to 5, and the number of threads to 1. The land-use transition matrix is presented in Table A2, wherein a value of 1 denotes a permissible transition, while a value of 0 indicates a prohibited transition.

The Kappa coefficient (Kappa) is utilized to quantify the consistency between predicted outcomes and observed data, serving as a standard metric for evaluating the overall accuracy of simulated imagery. It is generally accepted that a Kappa value below 0.75 indicates suboptimal simulation performance, whereas a value exceeding 0.75 signifies a high degree of consistency between the simulated and actual imagery, suggesting better simulation efficacy(van Vliet et al., 2011). In this study, the land-use simulation for the year 2020 yielded a Kappa coefficient of 0.959 and an overall accuracy of 97.46%. These metrics demonstrated that the model had an excellent performance in projecting land-use patterns in 2050.

**Table A1** The land-use demand in 2050 (grids number) and neighborhood weights for each land-use type.

| Land-use types | Cultivated land | Forestland | Grassland | Waters | Construction land | Unused land |
| --- | --- | --- | --- | --- | --- | --- |
| Land-use demand | 12838 | 10645 | 72060 | 4264 | 1267 | 39070 |
| Neighborhood weights | 1.000000 | 0.444444 | 0.134703 | 0.573820 | 0.527397 | 0 |

**Table A2** The land-use transition matrix in 2050

| The initial  Land-use types | The simulated land-use types | | | | | |
| --- | --- | --- | --- | --- | --- | --- |
|  | Cultivated land | Forestland | Grassland | Waters | Construction land | Unused land |
| Cultivated land | 1 | 1 | 1 | 0 | 1 | 1 |
| Forestland | 1 | 1 | 1 | 0 | 1 | 1 |
| Grassland | 1 | 1 | 1 | 0 | 1 | 1 |
| Waters | 1 | 1 | 1 | 1 | 1 | 1 |
| Construction land | 0 | 0 | 0 | 0 | 1 | 0 |
| Unused land | 1 | 1 | 1 | 0 | 0 | 1 |

**References**

Liang, X., Guan, Q., Clarke, K.C., Liu, S., Wang, B., Yao, Y., 2021. Understanding the drivers of sustainable land expansion using a patch-generating land use simulation (PLUS) model: A case study in Wuhan, China. Computers, Environment and Urban Systems 85, 101569. https://doi.org/10.1016/j.compenvurbsys.2020.101569

van Vliet, J., Bregt, A.K., Hagen-Zanker, A., 2011. Revisiting Kappa to account for change in the accuracy assessment of land-use change models. Ecological Modelling 222, 1367–1375. https://doi.org/10.1016/j.ecolmodel.2011.01.017

Wang, J., Zhang, J., Xiong, N., Liang, B., Wang, Z., Cressey, E.L., 2022. Spatial and Temporal Variation, Simulation and Prediction of Land Use in Ecological Conservation Area of Western Beijing. Remote Sensing 14, 1452. https://doi.org/10.3390/rs14061452

Wang, Q., Guan, Q., Sun, Y., Du, Q., Xiao, X., Luo, H., Zhang, J., Mi, J., 2023. Simulation of future land use/cover change (LUCC) in typical watersheds of arid regions under multiple scenarios. Journal of Environmental Management 335, 117543. https://doi.org/10.1016/j.jenvman.2023.117543

Xu, L., Liu, X., Tong, D., Liu, Z., Yin, L., Zheng, W., 2022. Forecasting Urban Land Use Change Based on Cellular Automata and the PLUS Model. Land 11, 652. https://doi.org/10.3390/land11050652
